# Identification of MKRN1 as a second E3 ligase for Eag1 potassium channels reveals regulation *via* differential degradation

**DOI:** 10.1016/j.jbc.2021.100484

**Published:** 2021-02-27

**Authors:** Ya-Ching Fang, Ssu-Ju Fu, Po-Hao Hsu, Pei-Tzu Chang, Jing-Jia Huang, Yi-Chih Chiu, Yi-Fan Liao, Guey-Mei Jow, Chih-Yung Tang, Chung-Jiuan Jeng

**Affiliations:** 1 Institute of Anatomy and Cell Biology, School of Medicine, National Yang-Ming University, Taipei, Taiwan; 2 Department of Physiology, College of Medicine, National Taiwan University, Taipei, Taiwan; 3 School of Medicine, Fu-Jen Catholic University, New Taipei City, Taiwan; 4 Brain Research Center, National Yang-Ming University, Taipei, Taiwan

**Keywords:** ubiquitin ligase, protein degradation, potassium channel, ER-associated degradation, ER quality control, glycosylation, homeostasis, BFA, brefeldin A, C, carboxyl, CHX, cycloheximide, CNBHD, cyclic nucleotide-binding homology domain, CQ, chloroquine, CUL7, cullin 7, DIV, day *in vitro*, Eag1-QQ, Eag1 N388Q-N406Q mutant, Endo H, endoglycosidase H, ER, endoplasmic reticulum, GST, glutathione S-transferase, HEK, human embryonic kidney, MKRN1, makorin ring finger protein 1, MKRN1-S, MKRN1 short isoform, N, amino, N-linked, asparagine-linked, nano-LC-MS/MS, nanoscale liquid chromatography coupled to tandem mass spectrometry, PLA, proximity ligation assay, PMSF, phenylmethylsulfonyl fluoride, PNGase F, N-glycosidase F, shRNA, short hairpin RNA, siRNA, small interference RNA, TMBTS, Temple–Baraitser syndrome, Ub, ubiquitin, VEGFR-2, vascular endothelial growth factor receptor-2, WT, wild-type, ZLS, Zimmermann–Laband syndrome

## Abstract

Mutations in the human gene encoding the neuron-specific Eag1 voltage-gated K^+^ channel are associated with neurodevelopmental diseases, indicating an important role of Eag1 during brain development. A disease-causing Eag1 mutation is linked to decreased protein stability that involves enhanced protein degradation by the E3 ubiquitin ligase cullin 7 (CUL7). The general mechanisms governing protein homeostasis of plasma membrane- and endoplasmic reticulum (ER)-localized Eag1 K^+^ channels, however, remain unclear. By using yeast two-hybrid screening, we identified another E3 ubiquitin ligase, makorin ring finger protein 1 (MKRN1), as a novel binding partner primarily interacting with the carboxyl-terminal region of Eag1. MKRN1 mainly interacts with ER-localized immature core-glycosylated, as well as nascent nonglycosylated, Eag1 proteins. MKRN1 promotes polyubiquitination and ER-associated proteasomal degradation of immature Eag1 proteins. Although both CUL7 and MKRN1 contribute to ER quality control of immature core-glycosylated Eag1 proteins, MKRN1, but not CUL7, associates with and promotes degradation of nascent, nonglycosylated Eag1 proteins at the ER. In direct contrast to the role of CUL7 in regulating both ER and peripheral quality controls of Eag1, MKRN1 is exclusively responsible for the early stage of Eag1 maturation at the ER. We further demonstrated that both CUL7 and MKRN1 contribute to protein quality control of additional disease-causing Eag1 mutants associated with defective protein homeostasis. Our data suggest that the presence of this dual ubiquitination system differentially maintains Eag1 protein homeostasis and may ensure efficient removal of disease-associated misfolded Eag1 mutant channels.

The *ether-à-go-go* family of voltage-gated K^+^ channels comprises three gene subfamilies: *eag* (*K*_*V*_*10*), *erg* (*eag*-related gene) (K_V_11), and *elk* (*eag*-like K^+^ channel) (K_V_12) (1). In mammals, Eag encodes neuron-specific K^+^ channels that are expressed in various regions of the brain and make significant contributions to subthreshold K^+^ currents; nonetheless, the specific physiological roles of Eag K^+^ channels are not yet fully understood (2, 3, 4, 5). The mammalian Eag subfamily encompasses two isoforms: Eag1 (K_V_10.1) and Eag2 (K_V_10.2) (3, 5, 6, 7, 8). Both *in situ* hybridization and real-time PCR analyses support the notion that Eag1 and Eag2 are highly expressed in a wide variety of different rat brain areas (2, 5, 9, 10), implying that the two mammalian Eag isoforms may participate in certain essential functions in the brain.

In *Drosophila*, mutations in the *eag* gene manifest a hyperexcitable phenotype in which fly legs shake extensively under ether anesthesia (11). Knockout of the Eag1 channel function in mice, however, fails to result in any marked phenotype other than a modest synaptic hyperactivity (12). Interestingly, knockdown of Eag1 in zebrafish reveals a severe disruption in the development of the central nervous system (13). Consistent with the observation in zebrafish, mutations in the human gene encoding Eag1 (*KCNH1*) were recently reported in individuals with two congenital neurodevelopmental disorders: Temple–Baraitser syndrome (TMBTS) (14) and Zimmermann–Laband syndrome (ZLS) (15, 16). TMBTS is characterized by intellectual disability, epilepsy, dysmorphic facial features, and broad thumbs and great toes with absent/hypoplastic nails; whereas ZLS is characterized by facial dysmorphism including coarsening of the face and a large nose, gingival enlargement, intellectual disability, hypoplasia of terminal phalanges and nails, and hypertrichosis. This correlation between *KCNH1* mutations and the clinical presentation of TMBTS/ZLS highlights a potentially important role of Eag1 during the development of the brain.

We have previously demonstrated that a ZLS-causing mutant Eag1 channel (G469R) is associated with significant reduction in total protein level (17), suggesting the presence of a disrupted Eag1 protein homeostasis. Protein homeostasis (also known as proteostasis) refers to the property of a subcellular system that regulates and maintains a stable balance of protein synthesis and degradation (18). Protein homeostatic regulation plays a critical role in determining the optimal expression level of endogenous K^+^ channels in neurons, which in turn may set the network excitability in a wide variety of different neural circuits in the brain (19). Very little is known about the molecular regulation of Eag1 protein homeostasis. Previously, we reported that the E3 ubiquitin ligase cullin 7 (CUL7) promotes proteasomal and lysosomal degradation of endoplasmic reticulum (ER)- and plasma membrane-localized Eag1 proteins, respectively (17). In this study, we present the identification of another novel binding partner of Eag1, the E3 ubiquitin ligase makorin ring finger protein 1 (MKRN1; also known as RNF61). The results from our biochemical and biophysical analyses are consistent with the idea that MKRN1 contributes to protein quality control of Eag1 K^+^ channels, as well as ER-associated degradation of disease-causing misfolded Eag1 proteins.

## Results

### MKRN1 is a novel binding partner of Eag1

To search for the protein machinery responsible for homeostatic regulation of the Eag1 K^+^ channel, we carried out yeast two-hybrid screening of a rat brain cDNA library by using the cytoplasmic carboxyl (C)-terminal region of rat Eag1 as the bait. Two of the positive clones isolated by the screening correspond to MKRN1, which belongs to the RING E3 ubiquitin ligase family and contains both the RING-finger E2-binding domain and the substrate-binding domain in the same protein (20). In addition to regulating protein homeostasis, MKRN1 may also interact with RNA-binding proteins and contribute to mRNA quality control (21, 22). There are two MKRN1 isoforms, the full-length, long isoform (isoform 1; 482 amino acids in human; 481 amino acids in rat) and the C-terminal truncated, short isoform (MKRN1-S; isoform 2; 329 amino acids in both human and rat), both of which are abundantly expressed in the brain (20, 23, 24). Importantly, only the long isoform of MKRN1 contains the complete structural domains required for the E3 ubiquitin ligase function (24).

**Figure 1:**
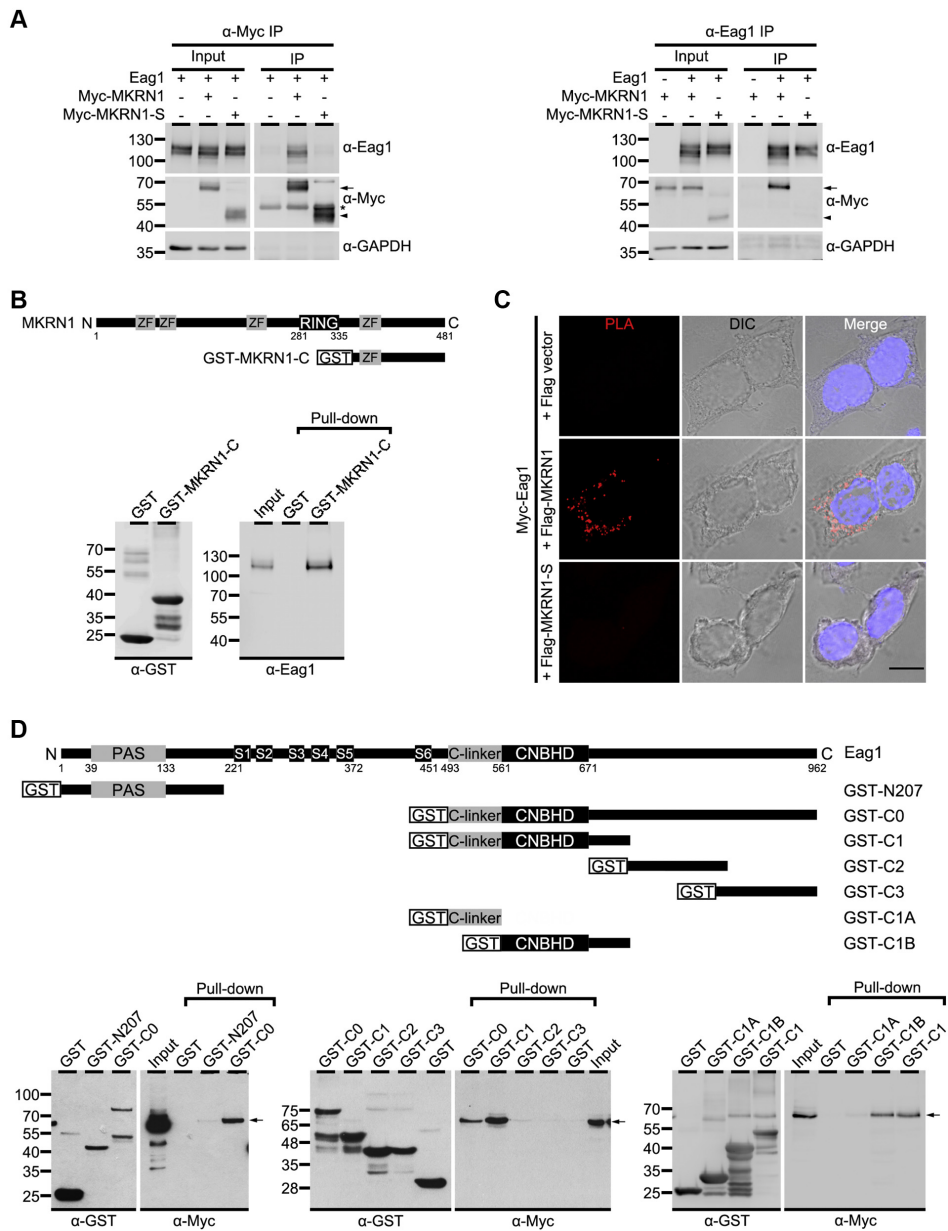
**The MKRN1 long isoform binds to Eag1.***A*, co-immunoprecipitation of Eag1 and MKRN1 in HEK293T cells. Lysates from HEK293T cells coexpressing rat Eag1 with the Myc vector, Myc-tagged MKRN1 long isoform (Myc-MKRN1), or Myc-tagged MKRN1 short isoform (Myc-MKRN1-S) were immunoprecipitated (IP) with the anti-Myc (α-Myc) (*left*) or anti-Eag1 (α-Eag1) (*right*) antibodies, followed by immunoblotting with the α-Myc, α-Eag1, or anti-GAPDH (α-GAPDH) antibodies. Corresponding expression levels of Eag1 and Myc-MKRN1 in the lysates are shown in the *Input* lane. Henceforward, input volumes correspond to 5% of the total cell lysates used for immunoprecipitation. The positions of molecular mass markers (in kilodalton, kDa) are indicated to the left. The protein bands corresponding to Myc-MKRN1 or Myc-MKRN1-S are highlighted with *arrow* and *arrowhead*, respectively. *Asterisk* indicates IgG heavy chain. The expression of GAPDH was used as the loading control. *B*, GST pull-down assay with rat brain lysates. *Top*, schematic representation of the GST-MKRN1-C1 fusion protein (encoding MKRN1 amino acids 355–481). *Bottom*, representative immunoblot. Brain lysates were subject to the pull-down assay with the GST protein or the GST-MKRN1-C fusion protein, followed by immunoblotting with the anti-GST (α-GST) or α-Eag1 antibodies. *C*, representative confocal micrographs for *in situ* proximity ligation assay (PLA) in HEK293T cells coexpressing Myc-Eag1 with the Flag vector, Flag-MKRN1, or Flag-MKRN1-S. Fixed cells were stained with both α-Myc and α-Flag under the permeabilized configuration. In all conditions, a pair of oligonucleotide-linked Duolink PLA secondary antibodies was applied. The fluorescence signal (*red*) was only detected when the oligonucleotide labeled α-Myc and α-Flag were in close proximity. Cells were also stained with the nuclear counterstain DAPI (*blue*; *right panels*). Cells were imaged in both the fluorescence (*PLA*; *left panels*) and the differential interference contrast (*DIC*; *center panels*) modes. *D*, GST pull-down assay with HEK293T lysates. *Top*, structural topology of the Eag1 channel and the GST-Eag1 fusion proteins. In Eag1, the cytoplasmic N-terminal region contains a Per-Arnt-Sim (PAS) domain, and the cytoplasmic C-terminal region includes three structural domains: the carboxyl linker (C-linker), the cyclic nucleotide-binding homology domain (CNBHD), and the post-CNBHD region. The amino acid range of various GST-Eag1 fusion proteins: GST-N207 (1–207), GST-C0 (493–962), GST-C1 (493–724), GST-C2 (723–848), GST-C3 (835–962), GST-C1A (493–560), GST-C1B (561–722). *Bottom*, representative immunoblots. Cell lysates prepared from HEK293T cells expressing Myc-MKRN1 were used for GST pull-down assay with GST or the indicated GST-Eag1 fusion proteins, followed by immunoblotting with α-Myc or α-GST. *Arrows* denote Myc-MKRN1 protein bands.

The protein sequences of the two rat MKRN1 clones isolated by yeast two-hybrid screening correspond to the partial segments (amino acids 142–481 and 355–481) of the long isoform and are virtually absent in the short isoform, suggesting that Eag1 may mainly interact with the MKRN1 long isoform. To address this possibility, we went on to perform the co-immunoprecipitation experiment. Rat Eag1 was coexpressed with either Myc-tagged rat MKRN1 long isoform (Myc-MKRN1) or Myc-tagged rat MKRN1 short isoform (Myc-MKRN1-S) in HEK293T cells. By employing both the anti-Myc and the anti-Eag1 antibodies for immunoprecipitation, we demonstrate in Figure 1*A* that only the long isoform of MKRN1, but not the short isoform, was co-immunoprecipitated with Eag1. We also generated a GST fusion protein encoding the C-terminal region (amino acids 355–481) of the MKRN1 long isoform (GST-MKRN1-C) and performed GST pull-down assay using rat brain lysates. As shown in Figure 1*B*, endogenous Eag1 protein in the brain lysates was readily precipitated by GST-MKRN1-C. Additionally, endogenous MKRN1 and Eag1 coexist in the same protein complex in neurons (Fig. S1). The direct interaction between Eag1 and MKRN1 long isoform, but not the short isoform, in HEK293T cells was further validated by the *in situ* proximity ligation assay (PLA) (Fig. 1*C*), which detects immunofluorescence signals generated from a pair of oligonucleotide-linked antibodies recognizing two proteins that are in close proximity (<40 nm) (25, 26, 27). Together these observations indicate that Eag1 interacts primarily with the C-terminal region of the long isoform of MKRN1.

The bait sequence used for screening the rat brain cDNA library corresponds to the cytoplasmic C-terminal region (amino acids 493–962) of rat Eag1, suggesting that MKRN1 probably interacts with the C-terminal region of Eag1. To test this hypothesis, we generated GST fusion proteins encoding either the amino (N)-terminal (GST-N207) or the C-terminal (GST-C0) region of rat Eag1 (Fig. 1*D*). Indeed, MKRN1 was effectively pulled down by the GST-Eag1 fusion protein GST-C0, but not by GST-N207. To further locate the MKRN1-interacting domain in Eag1, we generated three additional GST fusion proteins containing different Eag1 C-terminal domains: GST-C1 (amino acids 493–724), GST-C2 (amino acids 723–848), and GST-C3 (amino acids 835–962) (Fig. 1*D*). MKRN1 was efficiently pulled down by GST-C1, but not by GST-C2 and GST-C3, indicating that the proximal C-terminal region of Eag1 harbors the major MKRN1-interacting domain. The proximal C-terminal region of Eag1 contains a C-linker domain and a cyclic nucleotide-binding homology domain (CNBHD). We therefore made two additional GST fusion proteins that encode either the C-linker (GST-C1A: amino acid 493–560) or the CNBHD (GST-C1B: amino acids 561–722) region. As depicted in Figure 1*D*, MKRN1 preferentially binds to GST-C1B, implying that MKRN1 may directly interact with the Eag1 CNBHD.

### MKRN1 preferentially interacts with immature Eag1

Upon close inspection of the PLA immunofluorescence signals of Eag1 and MKRN1 long isoform (Fig. 1*C*), which probably reflect the direct interaction of the two macromolecules, we noticed that the majority of the signals was localized at the cytoplasmic perinuclear region. Since properly folded, mature Eag1 proteins are generally considered plasma membrane ion channels, the foregoing PLA images appear to imply that MKRN1 mainly interacts with immature Eag1 proteins that either have yet to or even fail to reach the plasma membrane.

**Figure 2:**
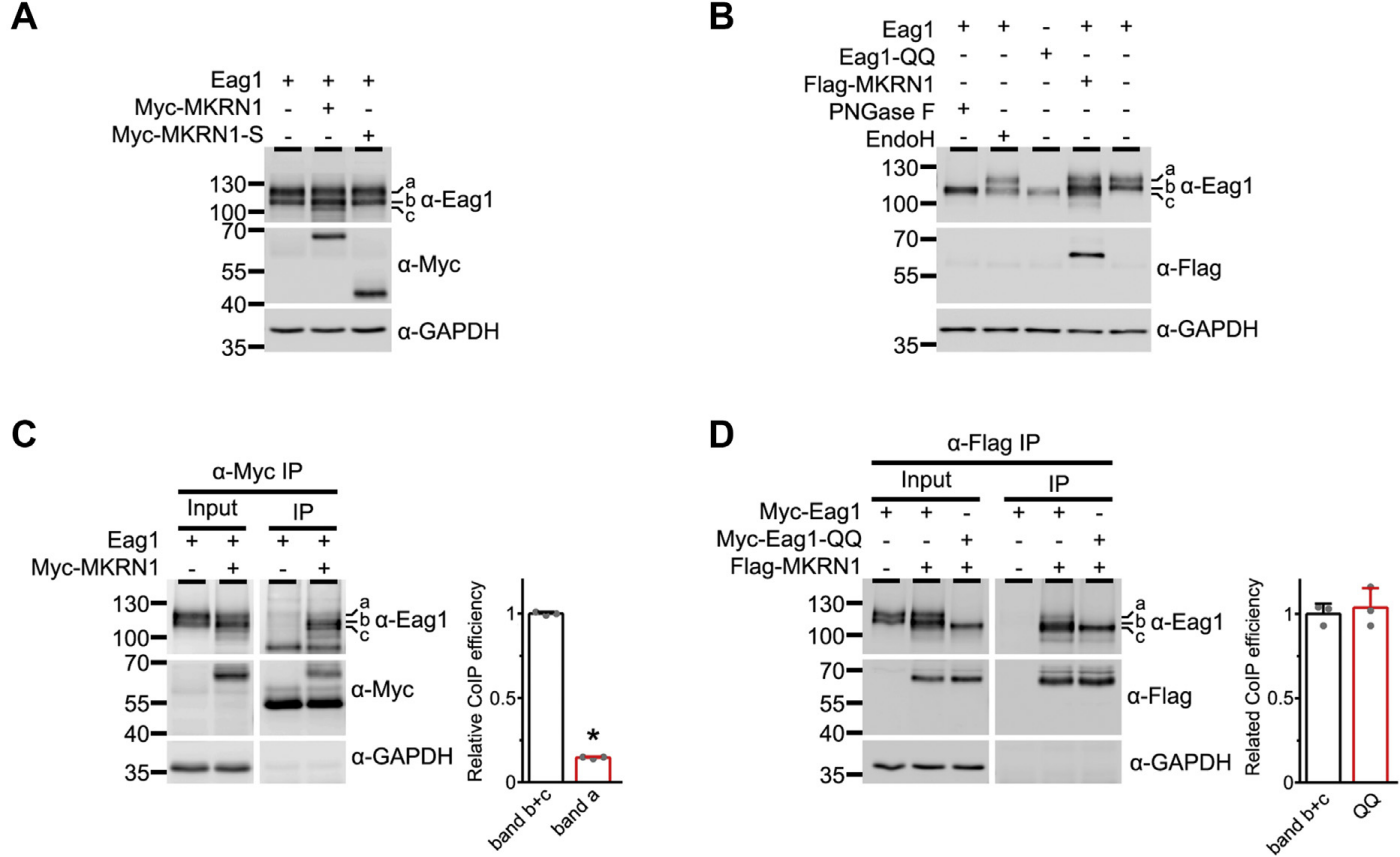
**MKRN1 interacts with immature Eag1.***A*, MKRN1 induces the presence of Eag1 protein band c. Eag1 was coexpressed with the Myc vector, Myc-MKRN1, or Myc-MKRN1-S in HEK293T cells. Eag1 expression manifests as three distinct protein bands (*a*, *b*, *c*) of 110 to 120 kDa. *B*, protein band c corresponds to nonglycosylated Eag1. Cell lysates prepared from HEK293T cells expressing Eag1 or the Eag1-glycosylation mutant (Eag1-QQ) were employed for deglycosylation assays with Endo H or PNGase F: “band a” is PNGase F-sensitive but Endo H-insensitive; “band b” is PNGase F- and Endo H-sensitive; “band c” shares the same apparent molecular weight with the Eag1-QQ mutant. *C*, MKRN1 displays higher binding affinity to immature Eag1. *Left*, representative co-immunoprecipitation (CoIP) data. Eag1 was coexpressed with the Myc vector or Myc-MKRN1 in HEK293T cells. Cell lysates were immunoprecipitated with α-Myc. *Right*, quantification of relative CoIP efficiency. Protein densities of IP mature and immature Eag1 signals were standardized by those of corresponding Input signals, followed by normalization with respect of immature Eag1 (band b+c) (∗*p* < 0.05; n = 3). *D*, MKRN1 binds to nonglycosylated Eag1. *Left*, representative CoIP data. Myc-tagged Eag1 or Eag1-QQ was coexpressed with Flag-tagged MKRN1 in HEK293T cells. Cell lysates were immunoprecipitated with α-Flag. *Right*, quantification of relative CoIP efficiency. Data were normalized with respect to immature Eag1 (band b+c) (n = 3).

To address this interesting possibility, we studied the effect of MKRN1 on Eag1 protein maturation in HEK293T cells. Eag1 harbors two consensus asparagine (N)-linked glycosylation sites (N388 and N406) (28). Importantly, the electrophoretic mobility pattern of Eag1 proteins on an immunoblot typically manifests as two major protein bands: i) a slower (high-molecular-weight) band “a” that corresponds to mature, full-glycosylated Eag1 proteins localized at the plasma membrane, and ii) a faster (low-molecular-weight) band “b” that represents immature, core-glycosylated Eag1 proteins at the ER (Fig. 2*A*) (17). By coexpressing Eag1 with MKRN1, we noticed the presence of another Eag1-associated low-molecular-weight protein band “c” (Fig. 2*A*). In contrast, no significant band c was detected when Eag1 was coexpressed with the noninteracting MKRN1-S (Fig. 2*A*). Since the apparent electrophoretic mobility of the MKRN1-induced Eag1 band c is significantly faster than that of immature, core-glycosylated Eag1 proteins (band b), it is possible that band c may correspond to nascent, nonglycosylated Eag1 proteins. To address this hypothesis, we employed an Eag1 mutant with the two consensus glycosylation asparagine residues mutated into glutamine (N388Q and N406Q; Eag1-QQ). Figure 2*B* shows that removing all N-linked oligosaccharides of Eag1 (both bands a and b) with the amidase PNGase F generates low-molecular-weight protein that appears to comigrate with the nonglycosylated Eag1-QQ mutant. Furthermore, cleaving high mannose oligosaccharides from immature, core-glycosylated Eag1 (band b) with Endo H also results in the presence of the Eag1-QQ-like, low-molecular-weight protein band (Fig. 2*B*). Mass spectrometry analyses of bands a, b, and c, as well as the single, deglycosylated band in response to PNGase F treatment, reveal that they indeed represent Eag1 proteins of distinct apparent molecular weights (Fig. S2; Table S1). Most importantly, all the above-mentioned nonglycosylated Eag1 protein bands share virtually identical electrophoretic mobility pattern with the MKRN1-induced Eag1 band c, consistent with the idea that band c represents nascent, nonglycosylated Eag1 proteins.

How can MKRN1 enhance the presence of nascent, nonglycosylated Eag1 proteins? One possibility is that MKRN1 may promote the deglycosylation of core-glycosylated Eag1 and/or prevent the glycosylation of nascent Eag1. If this inference is true, MKRN1 would be expected to display a differential binding affinity between immature Eag1 (bands b and c) and mature Eag1 (band a). Indeed, Figure 2*C* illustrates that, compared with their mature counterpart, immature Eag1 proteins were more efficiently co-immunoprecipitated with MKRN1. In contrast, no discernible difference in the co-immunoprecipitation efficiency with MKRN1 was detected between immature Eag1 proteins (bands b and c) and the nonglycosylated Eag1-QQ mutant (Fig. 2*D*). Taken together, the foregoing results support the notion that MKRN1 preferentially interacts with immature Eag1 proteins that are probably located at the cytoplasm, such as the ER or the *cis*-Golgi apparatus.

### MKRN1 downregulates Eag1 protein level

**Figure 3:**
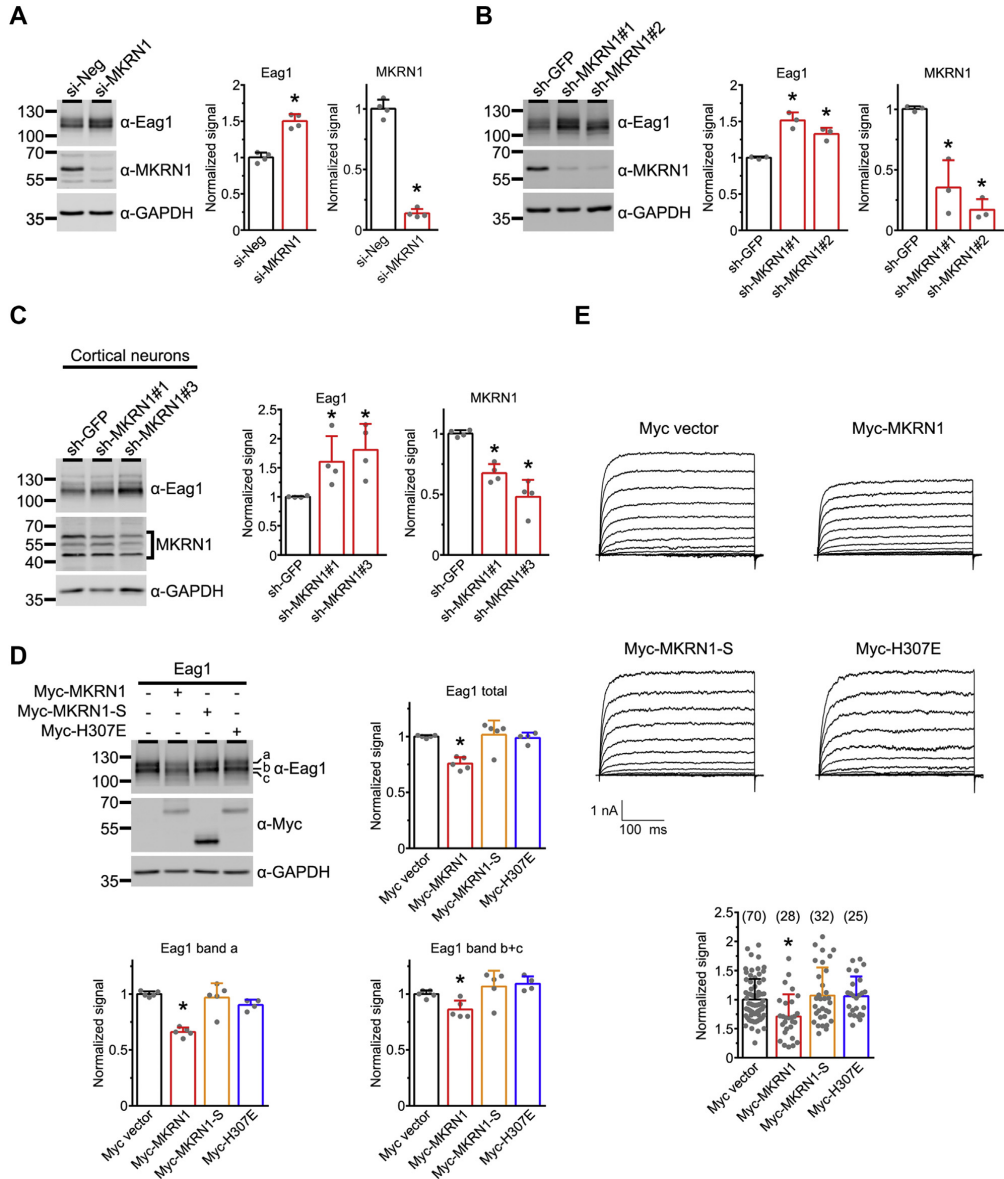
**MKRN1 downregulates Eag1 protein level.***A*, siRNA knockdown of endogenous MKRN1 in HEK293T cells. HEK293T cells overexpressing Eag1 were subject to treatment with a negative control siRNA (si-Neg), or a siRNA specific for MKRN1 (si-MKRN1). Quantitative analyses are summarized by the bar graphs. Protein densities were standardized as the ratio to the cognate GAPDH signals, followed by normalization with respect to the si-Neg control (∗*p* < 0.05; n = 4). *B*, shRNA knockdown of endogenous MKRN1 in HEK293T cells. HEK293T cells overexpressing Eag1 were subject to treatment with a control shRNA for GFP (sh-GFP), or two types of shRNA specific for MKRN1 (sh-MKRN1#1, sh-MKRN1#2). Quantitative analyses are based on normalization with respect to the sh-GFP control (∗*p* < 0.05; n = 4). *C*, shRNA knockdown of endogenous MKRN1 in cortical neurons. Cultured rat neurons were subject to treatment with a control shRNA for GFP (sh-GFP), or two types of shRNA specific for MKRN1 (sh-MKRN1#1, sh-MKRN1#3). Quantitative analyses are based on normalization with respect to the sh-GFP control (∗*p* < 0.05; n = 4). *D*, effect of MKRN1, MKRN1-S, or MKRN1-H307E overexpression on Eag1 protein level. Eag1 was cotransfected into HEK293T cells with the indicated constructs in the molar ratio 1:5. Quantitative analyses are based on normalization with respect to the Myc vector control (∗*p* < 0.05; n = 4–5). *E*, effect of MKRN1, MKRN1-S, or MKRN1-H307E overexpression on Eag1 current level. *Top*, representative Eag1 K^+^ current traces in the presence of the indicated constructs. The holding potential was −90 mV, and the pulse potentials ranged from −80 to +60 mV. *Bottom*, quantification of Eag1 whole-cell current density at +40 mV. Data were normalized with respect to the Myc vector control (∗*p* < 0.05). The numbers of observations are shown in parentheses.

Next we asked whether MKRN1 may regulate protein homeostasis of Eag1 K^+^ channels. Upon siRNA or shRNA knockdown of endogenous MKRN1 expression in HEK293T cells, we observed a sizeable upregulation of total Eag1 protein level (Fig. 3, *A* and *B*; Fig. S3). A similar Eag1 upregulation effect was also detected when we knocked down endogenous MKRN1 expression in cultured cortical neurons (Fig. 3*C*). Conversely, overexpression of MKRN1, but not the short isoform (MKRN1-S) or a catalytically inactive MKRN1 construct with a point mutation in the E3 ligase domain (MKRN1-H307E) (29), results in a substantial reduction of both immature (bands b and c) and mature (band a) Eag1 proteins in HEK293T cells (Fig. 3*D*). Similarly, only MKRN1, but not MKRN1-S and MKRN1-H307E, impairs the functional expression of Eag1 K^+^ currents in HEK293T cells (Fig. 3*E*). MKRN1 fails to discernibly affect the biophysical property of Eag1 K^+^ channels, consistent with the idea that the observed reduction in membrane K^+^ conductance primarily reflects a disruption of Eag1 protein homeostasis by MKRN1. Moreover, in agreement with the aforementioned interaction of MKRN1 with the nonglycosylated Eag1-QQ mutant, coexpression with MKRN1 considerably decreases Eag1-QQ protein level (Fig. S4*A*).

**Figure 4:**
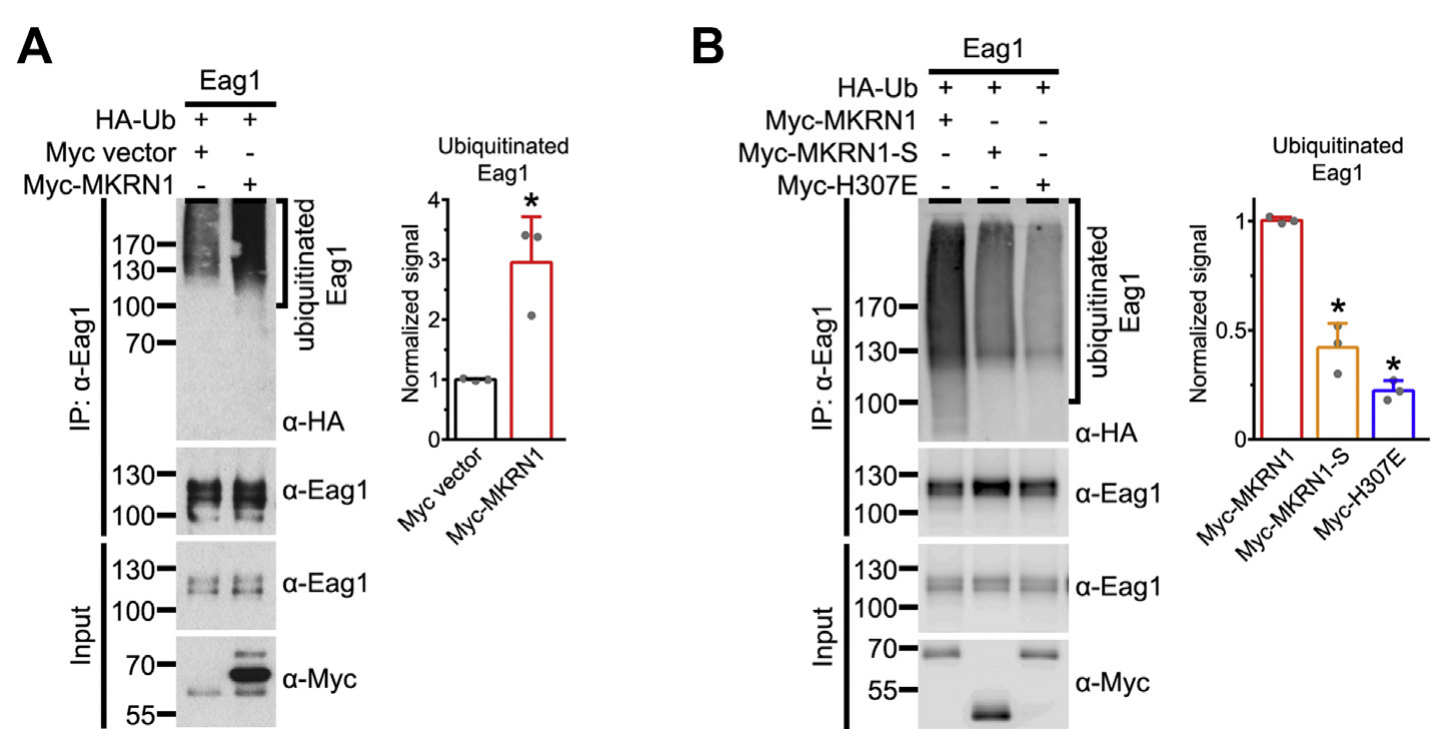
**MKRN1 promotes Eag1 ubiquitination.***A*, *Left*, representative immunoblot showing MKRN1-mediated Eag1 ubiquitination by HA-tagged ubiquitin (HA-Ub). Ubiquitinated Eag1 is visualized as protein smears with high molecular weights. *Right*, quantification of relative ubiquitinated Eag1 levels with respect to the Myc vector control (∗*p* < 0.05; n = 3). *B*, *Left*, representative immunoblots comparing the effect of MKRN1, MKRN1-S or MKRN1-H307E coexpression on Eag1 ubiquitination. *Right*, quantification of relative ubiquitinated Eag1 levels with respect to the effect of MKRN1 co-expression (∗*p* < 0.05; n = 3).

We then address the mechanism underlying the downregulation of Eag1 protein level by MKRN1. Semiquantitative RT-PCR analyses reveal that MKRN1 does not detectably affect the mRNA level of Eag1 (Fig. S5). This observation appears to rule out a potential transcriptional effect of MKRN1 on Eag1. Given its known role as a member of the RING finger E3 ubiquitin ligase superfamily (20), we also explored the possibility that MKRN1 may affect Eag1 protein homeostasis by catalyzing the ubiquitination of the K^+^ channel. Figure 4*A* illustrates that, in response to coexpression with HA-tagged ubiquitin (HA-Ub), basal Eag1 ubiquitination signal manifests as a faint, diffuse Eag1 protein smear conjugated with HA-Ub, consistent with the presence of Eag1 polyubiquitination in HEK293T cells. Importantly, overexpression of MKRN1 leads to a prominent enhancement of Eag1 polyubiquitination (Fig. 4*A*). Moreover, MKRN1 promotes polyubiquitination of the nonglycosylated Eag1-QQ mutant (Fig. S4*B*), further supporting the notion that glycosylation is not required for Eag1 regulation by MKRN1. In contrast, overexpression of either the short isoform MKRN1-S or the catalytically inactive mutant MKRN1-H307E fails to exert comparable ubiquitination-enhancing effect on Eag1 (Fig. 4*B*). Together these results suggest that MKRN1 effectively promotes Eag1 polyubiquitination, which leads to substantial downregulation of Eag1 protein level.

### MKRN1 enhances ER-associated degradation of immature Eag1

**Figure 5:**
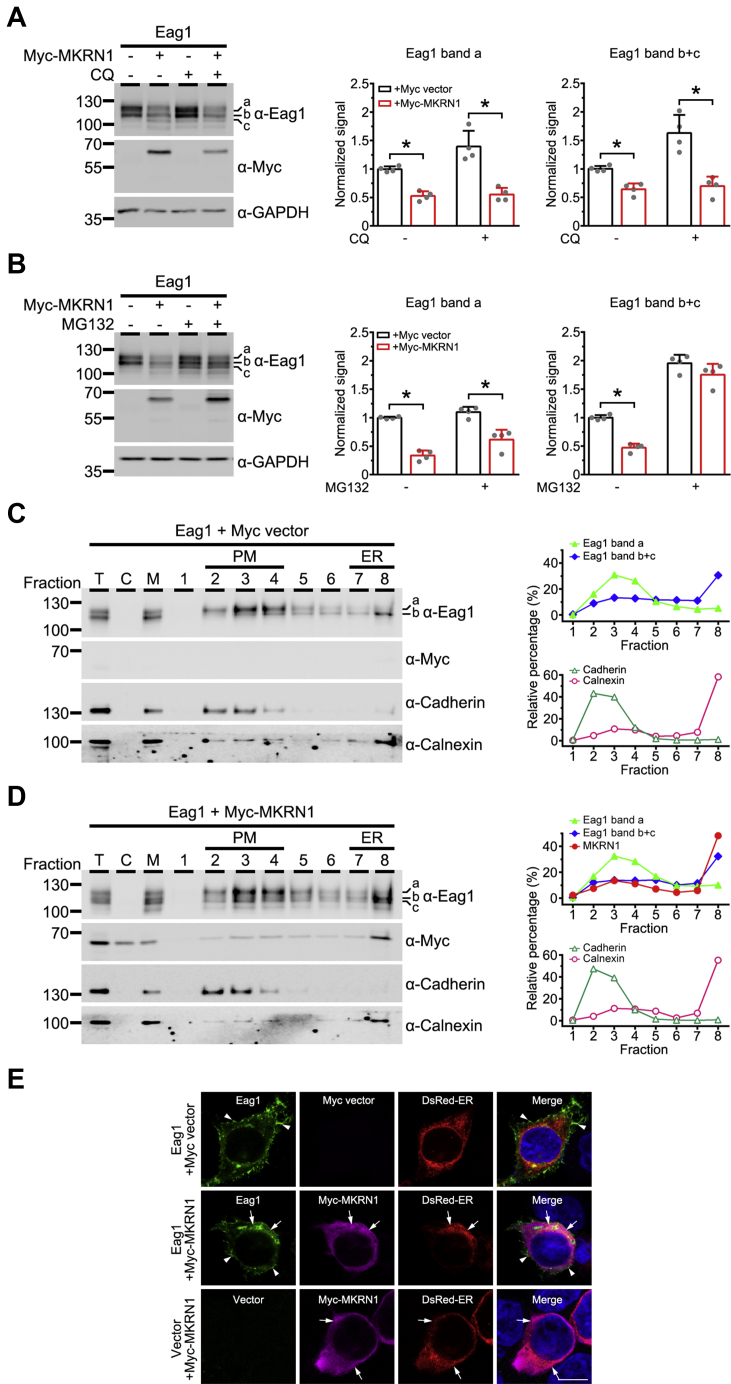
**MKRN1 mediates ER-associated proteasomal degradation of Eag1.***A* and *B*, effect of 10 μM chloroquine (CQ) or 10 μM MG132 treatment (in DMSO) on MKRN1 regulation of Eag1 protein levels in HEK293T cells. *Left panels*, representative immunoblots. *Right panels*, quantification of the effect of MKRN1 coexpression on mature (band a) or immature (band b+c) Eag1 protein levels in the absence or presence of drug treatment. Protein densities were standardized as the ratio to the cognate GAPDH signals, followed by normalization with respect to the corresponding Myc vector control (∗*p* < 0.05; n = 4). *C* and *D*, subcellular fractionation patterns of Eag1 in the absence or presence of Myc-MKRN1 coexpression in HEK293T cells. *Left panels*, representative immunoblots. Total cell homogenates (T) were ultracentrifuged and thereafter separated into the cytosol (C) and the membrane (M) fractions. The membrane pellet fraction was further sedimented through a discontinuous sucrose gradient and subsequently divided into eight fractions, with the density gradient increasing from fraction 1 toward fraction 8. Two endogenous proteins were used as specific markers for distinct subcellular compartments in HEK293T cells: cadherin (plasma membrane; fractions 2–4) and calnexin (ER membrane; fractions 7–8). *Right panels*, densitometric quantification of the relative distribution (with respect to the total signal) of the indicated protein bands in each membrane-associated fraction. *E*, representative immunofluorescence images showing the effect of MKRN1 (*magenta*) coexpression on the subcellular localization of Eag1 (*green*) in HEK293T cells. Nuclei were counterstained with DAPI (*blue*). Cells were cotransfected with the DsRed-ER expression vector (*DsRed-ER*) (*red*) to verify ER localization. *Arrowheads* indicate plasma membrane staining, whereas *arrows* denote intracellular staining. Scale bar, 10 μm. Data are representative of at least three independent experiments.

To ascertain the detailed mechanism of Eag1 downregulation by MKRN1, we went on to study the effect of two distinct types of protein degradation blockers, the lysosome inhibitor chloroquine (CQ) and the proteasome inhibitor MG132. Figure 5*A* demonstrates that, for both mature (band a) and immature (bands b and c) Eag1, the extent of protein downregulation by MKRN1 remains virtually the same in the absence or presence of CQ, suggesting against a mechanistic role of lysosomal degradation. Application of MG132 does not notably affect the downregulation of mature Eag1 by MKRN1 either (Fig. 5*B*). Nevertheless, MG132 treatment effectively abolishes the suppression effect of MKRN1 on the protein level of immature Eag1 (Fig. 5*B*). The selective rescue effect of MG132 on immature but not mature Eag1 appears to imply that MKRN1 efficiently promotes proteasomal degradation of immature Eag1. Furthermore, although MG132 markedly rescues MKRN1-promoted degradation of immature Eag1, the majority of the rescued immature Eag1 bands b and c may be substantially misfolded and is thus prevented from protein maturation into the mature Eag1 band a.

So far our data are consistent with the idea that MKRN1 preferentially interacts with and promotes degradation of immature Eag1, which suggests that both MKRN1 and immature Eag1 may be localized at the ER membrane. To directly address this hypothesis, we examined the subcellular localization of Eag1 and MKRN1 in HEK293T cells. Differential centrifugation analyses indicate that the ER-resident membrane protein calnexin, the plasma membrane protein cadherin, and Eag1 are exclusively present in the membrane fraction (Fig. 5*C*). Sucrose density gradient analyses further demonstrate that, in the absence of MKRN1, mature (band a) and immature (band b) Eag1 proteins were preferentially detected in the cadherin-enriched plasma membrane and the calnexin-enriched ER membrane fractions, respectively (Fig. 5*C*). In HEK293T cells, both endogenous and heterologously expressed MKRN1 display notable membrane association as well; moreover, in the presence of MKRN1, both immature Eag1 proteins (bands b and c) and MKRN1 are present in the calnexin-enriched ER membrane fraction (Fig. S6, *A* and *B*; Fig. 5*D*). Finally, confocal microscopic images illustrate that a significant portion of both Eag1 and MKRN1 immunofluorescence signals colocalize with the ER-targeting protein DsRed-ER, as well as exhibiting reticular perinuclear staining pattern (Fig. S6*C*; Fig. 5*E*).

**Figure 6:**
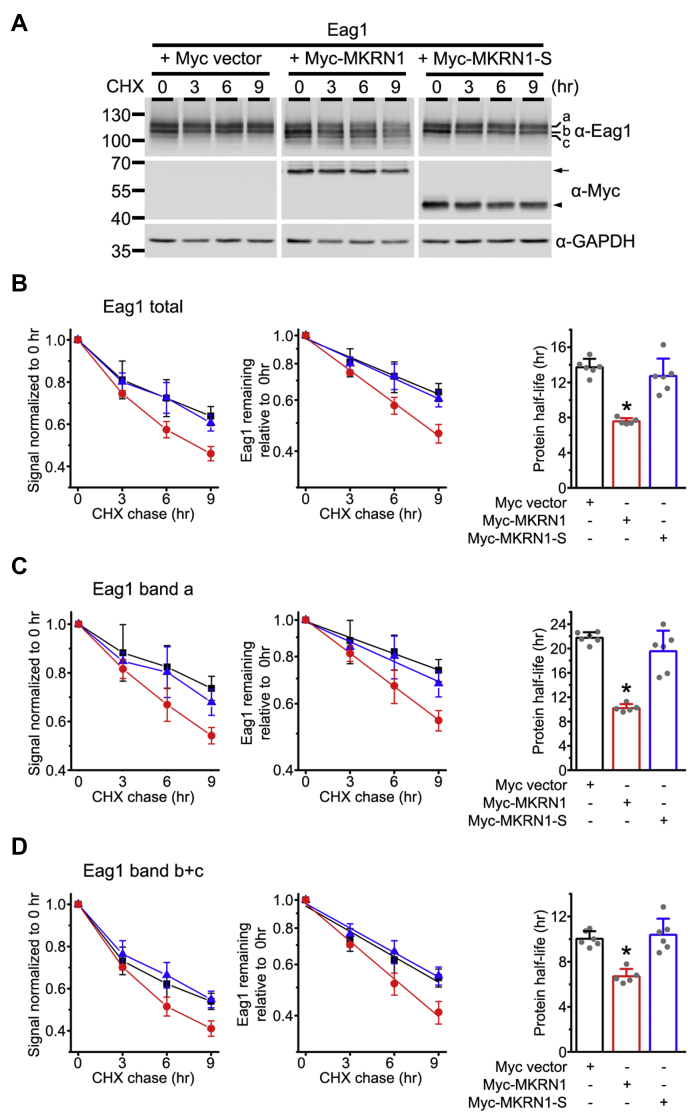
**MKRN1 reduces Eag1 protein stability.***A*, representative immunoblots showing the protein turnover time course of Eag1 coexpressed with the Myc vector, Myc-MKRN1, or Myc-MKRN1-S in HEK293T cells. Transfected cells were subject to 100 μg/ml cycloheximide (CHX) treatment for the indicated durations. *B*–*D*, quantification of the half-life values of Eag1 protein bands in response to different coexpression conditions. Symbol colors: Myc vector, *black*; MKRN1, *red*; MKRN1-S, *blue*. *Left panels*, linear plot of relative Eag1 protein band signals in response to different CHX treatment durations (based on the immunoblot shown in (*A*)). Protein densities were standardized as the ratio to the cognate GAPDH signals, followed by normalization with respect to the corresponding no treatment control at 0 h (*Center panels*) Linear-regression analyses (*solid lines*) of the semilogarithmic plot of the same data points shown to the *left*. *Right panels*, statistical analyses of Eag1 protein half-life values derived from multiple experiments (∗*p* < 0.05; n = 4–6).

Overall, the preceding observations support the notion that MKRN1 primarily promotes ER-associated proteasomal degradation of immature Eag1. To directly address whether MKRN1 affects the protein stability of Eag1, we applied the cycloheximide (CHX) chase assay to assess the effect of MKRN1 on Eag1 protein half-life. As shown in Figure 6, *A* and *B*, MKRN1 overexpression leads to about 45% reduction in the apparent half-life of total Eag1 protein, from about 13.7 h to about 7.6 h. In contrast, overexpression of the noninteracting MKRN1-S fails to discernibly change Eag1 total protein half-life (about 12.7 h). This result confirms that MKRN1 accelerates the protein turnover kinetics of Eag1.

In addition to total Eag1 protein, we also analyzed how MKRN1 affects protein stability of mature (band a) and immature (bands b and c) Eag1. Figure 6*C* depicts that, in the presence of MKRN1, the apparent protein half-life of mature Eag1 decreases by about 53%, from about 21.8 to 10.2 h. In addition, MKRN1 coexpression leads to about 33% diminution in apparent half-life of immature Eag1, from about 10.0 to 6.7 h (Fig. 6*D*). Given that MKRN1 preferentially interacts with immature Eag1, how can MKRN1 enhance protein turnover of mature Eag1?

**Figure 7:**
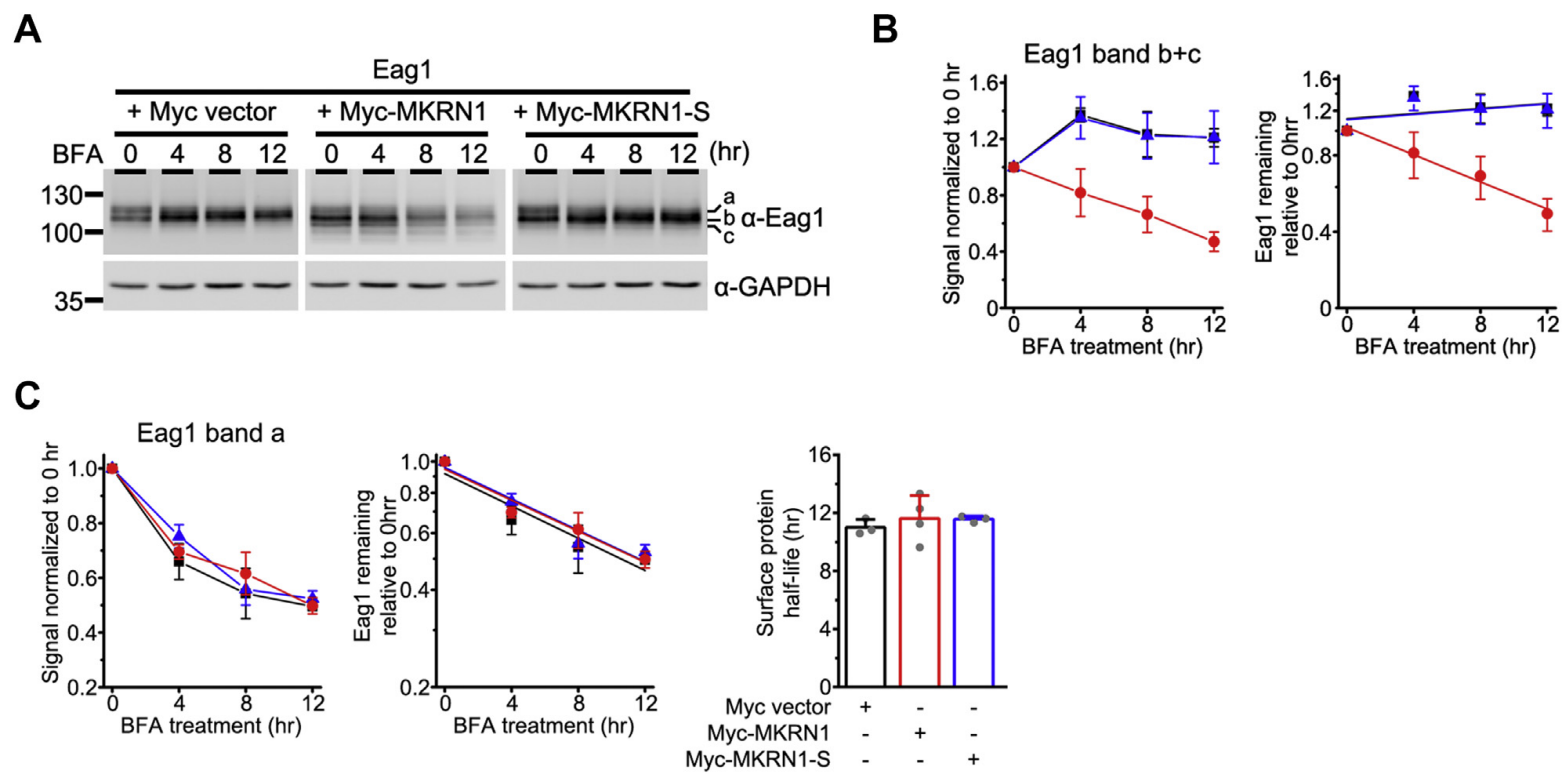
**MKRN1 fails to affect the protein stability of mature Eag1.***A*, representative immunoblots showing the effect of brefeldin A (BFA) treatment on mature and immature Eag1 protein bands. HEK293T cells coexpressing Eag1 and the indicated constructs were subject to up to 12 h of BFA treatment. *B*, MKRN1 reverses BFA-induced enhancement of immature (band b+c) Eag1 signals. *Left*, linear plot of relative immature Eag1 signals in response to different BFA treatment durations (based on the immunoblot shown in (*A*)). Protein densities were standardized as the ratio to the cognate GAPDH signals, followed by normalization with respect to the corresponding no treatment control at 0 h. Symbols: Myc vector, *black squares*; MKRN1, *red circles*; MKRN1-S, *blue triangles*. *Right*, linear-regression analyses (*solid lines*) of the semilogarithmic plot of the same data points shown to the *left*. *C*, MKRN1 does not affect the time course of BFA-induced reduction of mature (band a) Eag1 signals. *Left*, Linear plot of relative mature Eag1 signals in response to different BFA treatment durations (based on the immunoblot shown in (*A*)). Protein densities were standardized as the ratio to the cognate GAPDH signals, followed by normalization with respect to the corresponding no treatment control at 0 h. Symbols: Myc vector, *black squares*; MKRN1, *red circles*; MKRN1-S, *blue triangles*. *Center*, linear-regression analyses (*solid lines*) of the semilogarithmic plot of the same data points shown to the *left*. *Right*, Statistical analyses of mature (surface) Eag1 protein half-life values derived from multiple experiments (∗*p* < 0.05; n = 4).

The apparent protein half-life of mature Eag1, as resolved from the CHX chase assay in Figure 6, is in fact determined by the protein degradation kinetics of full-glycosylated mature Eag1 (band a) *per se*, as well as the protein maturation (*i.e.*, membrane trafficking) kinetics of core-glycosylated immature Eag1 (band b). In other words, the observed MKRN1-induced reduction in mature Eag1 half-life may reflect an enhanced degradation of mature Eag1 and/or a decreased membrane trafficking of immature Eag1. To differentiate these two possibilities, we employed the brefeldin A (BFA) chase assay to further investigate the effect of MKRN1 on mature Eag1 degradation at the cell surface (Fig. 7*A*). BFA obstructs the forward trafficking of proteins from the ER to the Golgi (30), and is therefore expected to block the conversion of Eag1 from its immature form (band b) into its mature form (band a), resulting in a time-dependent enrichment of immature Eag1 proteins (Fig. 7*B*). In response to MKRN1 (but not MKRN1-S) overexpression, however, immature Eag1 displays a time-dependent reduction in the BFA chase assay (Fig. 7*B*), consistent with the presence of MKRN1-induced enhanced degradation of immature Eag1. On the other hand, as a result of the BFA-induced blockade of forward membrane trafficking, the time-dependent decrease in the “band a” level in the BFA chase assay shall more faithfully reflect the degradation kinetics of mature Eag1 proteins. As illustrated in Figure 7*C*, in the absence of MKRN1, the apparent protein half-life of mature Eag1 is about 11.0 h. Most importantly, upon coexpression with either MKRN1 or MKRN1-S, the apparent protein half-life of mature Eag1 remains virtually unchanged (about 11.6 h), consistent with the idea that MKRN1 does not detectably affect the protein stability of mature Eag1 proteins at the cell surface. Therefore, the aforementioned effect of MKRN1 on the protein half-life of mature Eag1 predominantly reflects a time-dependent reduction in the amount of immature Eag1 trafficking to the plasma membrane.

### MKRN1 contributes to protein quality control of disease-causing Eag1 mutants

**Figure 8:**
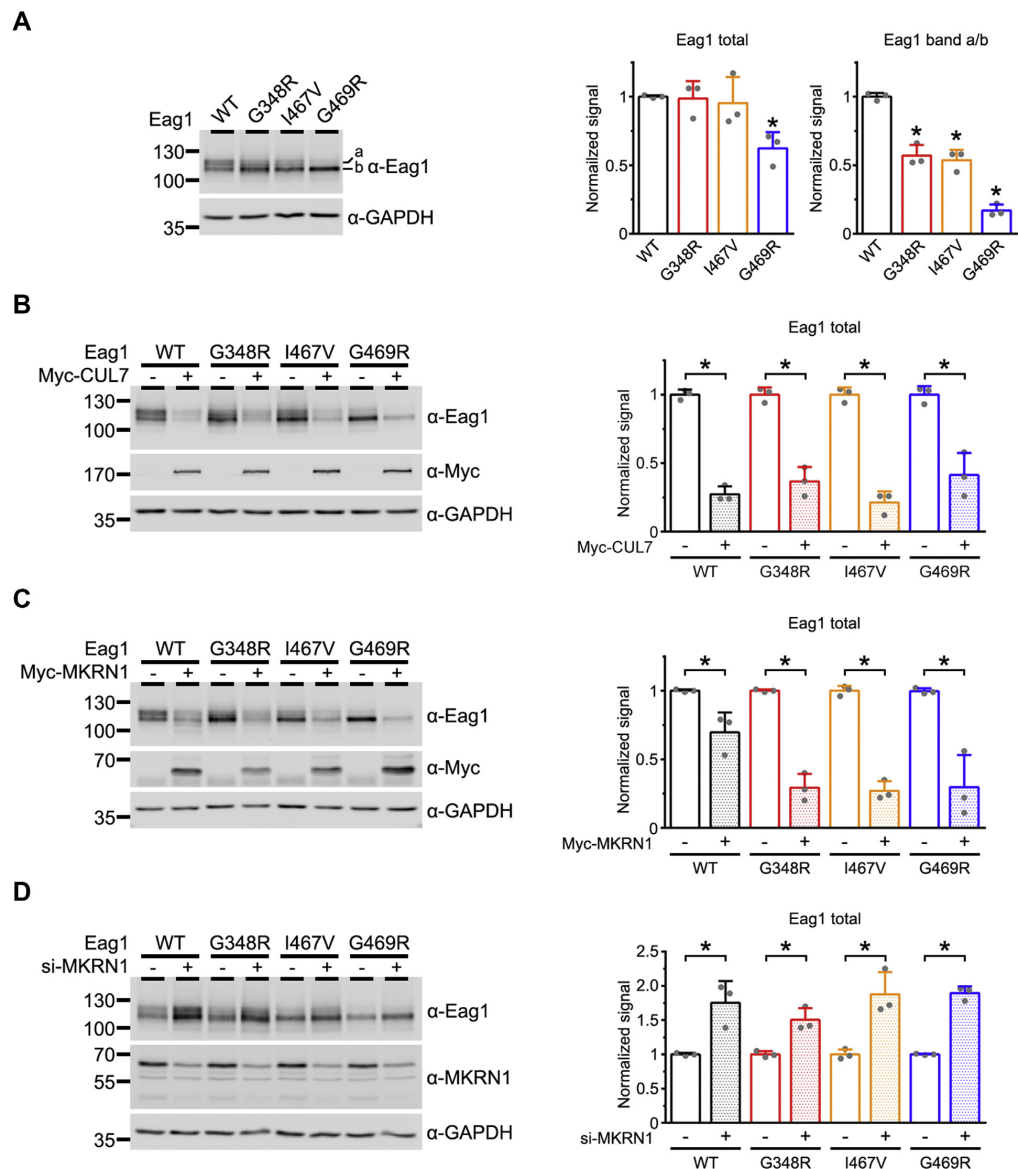
**MKRN1 regulates protein expression of disease-associated Eag1 mutants.***A*, *Left*, representative immunoblot comparing protein expression of Eag1 wild-type (WT), G348R, I467V, and G469R mutants in HEK293T cells. *Right*, statistical comparisons between WT and mutants were performed for total protein level (*Eag1 total*), as well as the relative signal ratio of band a to band b (*Eag1 band a/b*) (∗*p* < 0.05; n = 3). *B*–*D*, effect of CUL7 overexpression, MKRN1 overexpression, or siRNA knockdown of MKRN1 on WT and mutant Eag1 protein levels in HEK293T cells. *Left panels*, representative immunoblots. *Right panels*, quantification of relative Eag1 protein levels. Eag1 signals were standardized as the ratio to the cognate GAPDH signals, followed by normalization to the corresponding Myc-vector or siRNA control (∗*p* < 0.05; n = 3).

In human, the Eag1 K^+^ channel protein is encoded by the *KCNH1* gene. Several mutations in the *KCNH1* gene have been linked to the congenital neurodevelopmental anomalies TMBTS and ZLS (14, 15, 16). Previous functional analyses indicate that the ZLS-causing Eag1 mutations G348R and I467V give rise to functional K^+^ channels with hyperpolarizing shift of the steady-state voltage dependence property, but that another ZLS-causing Eag1 mutation, G469R, fails to generate detectable K^+^ currents (16). It remains unclear, however, whether these disease-causing mutations may affect Eag1 protein homeostasis. Figure 8*A* shows that, upon overexpression in HEK293T cells, the total protein level of the nonfunctional mutant G469R is substantially lower than that of the wild-type (WT) channel. In contrast, the total protein level of the two functional Eag1 mutants (G348R and I467V) is comparable with that of their WT counterpart. Nevertheless, we noticed that, relative to the WT, the band b signals of the mutants appear to be more prominent. We therefore compared the relative protein signal of band a (full-glycosylated form) to band b (core-glycosylated form) (band a/b ratio), which reflects the protein maturation efficiency of a given Eag1 construct. While the two functional Eag1 mutants display about 50% decrease in the band a/b ratio, the reduction level for the nonfunctional G469R mutant is more than 80% (Fig. 8*A*). Together, these observations are consistent with the idea that these ZLS-causing Eag1 mutants are associated with a significant defect in protein homeostasis.

Like the WT, the total protein level of the mutants was dramatically reduced in response to CUL7 or MKRN1 coexpression (Fig. 8, *B* and *C*). Moreover, similar to the effect of RNA interference of endogenous CUL7 expression (17), siRNA knockdown of endogenous MKRN1 in HEK293T cells leads to considerable upregulation of the total protein level of the mutants (Fig. 8*D*). Overall, our data indicate that both CUL7 and MKRN1 contribute to the protein quality control mechanism of the ZLS-causing Eag1 mutants.

## Discussion

A cardinal mechanism governing protein homeostasis of K^+^ channels involves exquisite posttranslational regulation of protein folding and degradation (19, 31). In this study, we identified MKRN1 as a novel binding partner of Eag1 K^+^ channels. MKRN1, a member of the RING E3 ubiquitin ligase family, contains the RING-finger E2-binding domain and the substrate-binding domain on the same polypeptide and is highly expressed in various mammalian cells (20, 23). Our biochemical analyses demonstrate that, *via* its C-terminal CNBHD region, Eag1 interacts primarily with the C-terminal segment of the long isoform of MKRN1. Importantly, we provided multiple lines of evidence suggesting that MKRN1 mainly interacts with ER-localized immature core-glycosylated, as well as nascent nonglycosylated, Eag1 proteins. Furthermore, MKRN1, but not the short isoform (MKRN1-S) or the catalytically inactive MKRN1 mutant (MKRN1-H307E), promotes polyubiquitination and proteasomal degradation of Eag1. Together these observations indicate that the long isoform of MKRN1 serves as an E3 ubiquitin ligase contributing to ER-associated proteasomal degradation of immature Eag1 proteins.

MKRN1 was initially reported to regulate cell differentiation and cell cycle by promoting human telomerase reverse transcriptase ubiquitination and proteasomal degradation (32, 33). MKRN1 was later demonstrated to play an essential role in controlling apoptosis, cell cycle arrest, and tumorigenesis *via* serving as the E3 ubiquitin ligase for such key signaling proteins as p21, p53, and phosphatase and tensin homolog (29, 34, 35, 36). The functions of other known substrate proteins for MKRN1 include viral shell protein, Wnt signaling, and cellular energy sensing (37, 38, 39). Therefore, to the best of our knowledge, here we present the first direct evidence indicating that MKRN1 may also affect neuronal membrane excitability by regulating protein homeostasis of the Eag1 channel.

An important step during the biogenesis of membrane proteins is the conversion of their glycosylation patterns from core (high mannose oligosaccharides) into mature (complex) glycans (40). The glycan composition of a membrane protein can therefore affect its folding, stability, and trafficking to the cell surface (41, 42). Previous studies have shown that Eag1 harbors two N-linked glycosylation sites, N388 and N406, and mutation at either of the two N-linked glycosylation sites results in prominent disruption of Eag1 protein stability and trafficking (28). We have demonstrated that inhibition of protein degradation by MG132 affects Eag1 glycosylation and trafficking to the surface (17), suggesting that constitutive ER clearance is essential for the transport of Eag1 channels to the membrane. Interestingly, suppression of Eag1 proteasomal degradation with MG132 results in the accumulation of a third protein band with the lowest apparent molecular weight (band c) that represents the nonglycosylated form of the K^+^ channel (Fig. 5*B*; Fig. S2) (17). Prior to proteasomal degradation, ubiquitinated glycoproteins are subject to retrotranslocation from the ER to the cytoplasm, followed by deglycosylation by N-glycanase (40, 43); therefore, the accumulated band c may correspond to MG132-rescued deglycosylated Eag1 in the cytoplasm. Alternatively, the MG132-induced band c may also represent nascent, nonglycosylated Eag1 localized at the ER. Coexpression with MKRN1, but not CUL7, leads to the accumulation of Eag1 band c as well (Fig. 2) (17). Importantly, sucrose density gradient analyses indicate that MKRN1 and Eag1 band c colocalize at the calnexin-enriched ER membrane fraction (Fig. 5), suggesting that MKRN1 interacts with nascent, nonglycosylated Eag1 at the ER, but not the cytosolic deglycosylated protein intermediate to be destructed by the proteasome. Provided that MKRN1 effectively promotes the ubiquitination of ER-localized core-glycosylated and nonglycosylated Eag1 proteins (Fig. 3), why does the E3 ubiquitin ligase induce the accumulation of nascent, nonglycosylated Eag1?

The E3 ubiquitin ligase HRD1 is responsible for ER-associated degradation of the ATP-binding cassette half-transporters ABCG8 and ABCG5, which form heterodimers at the ER before trafficking to the plasma membrane to control sterol balance (44, 45). In addition to the common E3 activity-dependent degradation mechanism, HRD1 may also inhibit ABCG8 glycosylation and thereby induce the accumulation of nonglycosylated ABCG8 at the ER. Moreover, RNF121, an ER-resident E3-ubiquitin ligase, regulates protein homeostasis of vascular endothelial growth factor receptor-2 (VEGFR-2) by preferentially interacting with newly synthesized, immature VEGFR-2 that is associated with lower protein stability, leading to an ubiquitin-mediated accumulation of immature VEGFR-2 at the ER (46). Taken together, we propose that, similar to HRD1 and RNF121, MKRN1 interacts primarily with misfolded core-glycosylated and nonglycosylated Eag1, thereby preventing the maturation of misfolded immature Eag1 proteins at the ER, as well as promoting their degradation in an E3 activity-dependent manner.

**Figure 9:**
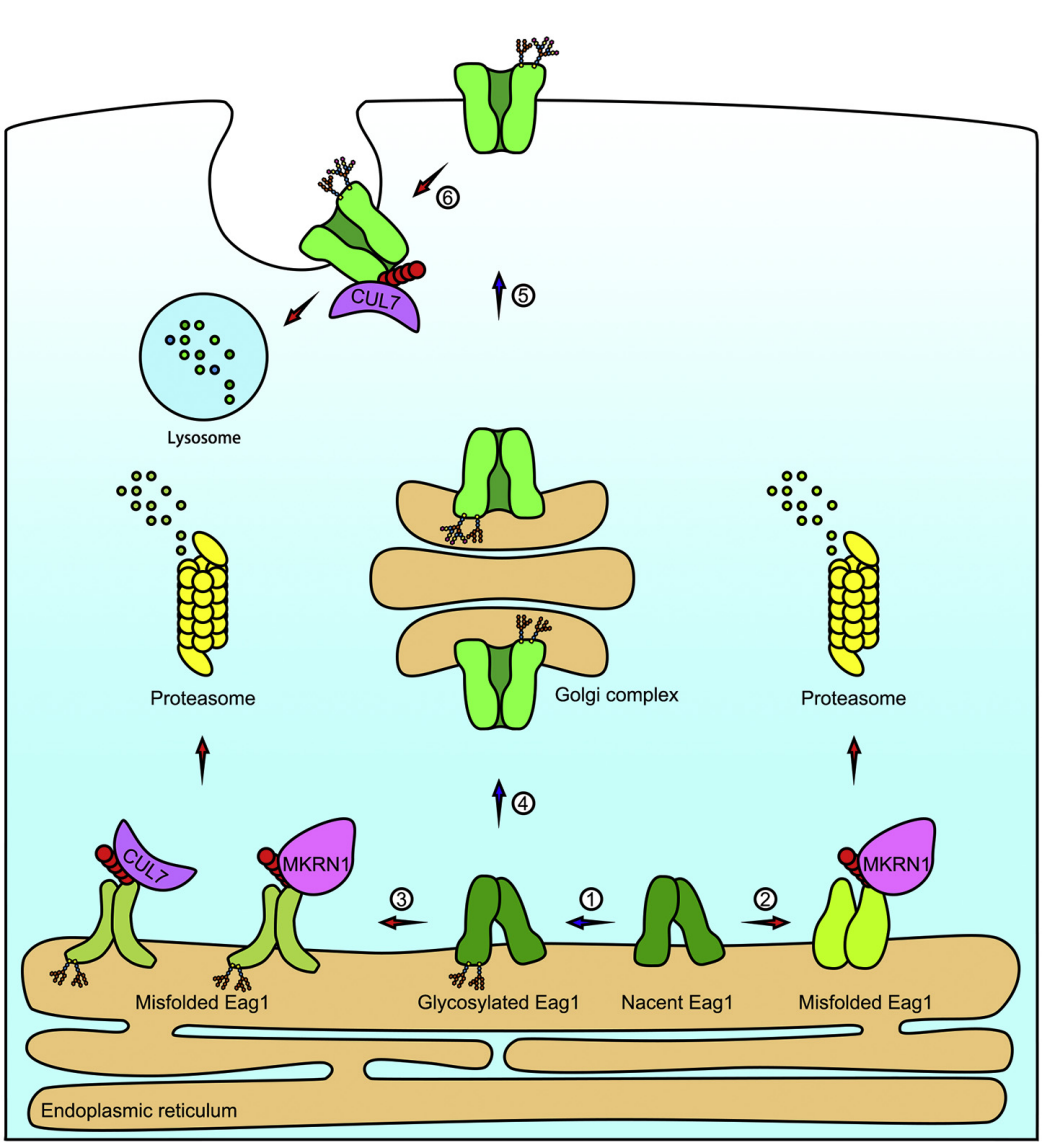
**A dual E3 ubiquitin ligase system mediates protein homeostasis of the Eag1 channel.** In this schematic model, we propose that two E3 ubiquitin ligases, CUL7 and MKRN1, play differential roles in the maintenance of Eag1 protein homeostasis. During biogenesis of immature Eag1 at the endoplasmic reticulum (ER), newly translated nascent, nonglycosylated Eag1 goes through initial glycosylation process in the ER lumen to form a core-glycosylated intermediate (pathway 1). Misfolded nonglycosylated Eag1 is recognized by MKRN1 and eventually subject to proteasomal degradation (pathway 2). Misfolded core-glycosylated Eag1, on the other hand, is identified by both CUL7 and MKRN1, followed by degradation through the ubiquitin-proteasome system (pathway 3). In contrast, properly folded immature Eag1 may exit the ER and undergo advanced glycan structure modification at the Golgi complex (pathway 4), followed by trafficking to the plasma membrane (pathway 5). At the membrane, misfolded or aged full-glycosylated Eag1 is subject to endosome-lysosomal degradation *via* a CUL7-depdendent mechanism (pathway 6). Overall, while both CUL7 and MKRN1 contribute to ER quality control and prevent ER exit of misfolded immature Eag1, CUL7 is additionally in charge of peripheral quality control of mature Eag1 at the cell surface.

Protein quality control, one of the most important forms of homeostatic regulation, involves rigorous molecular surveillances of protein folding conformations, as well as efficient targeting of misfolded or damaged proteins for degradation (47, 48, 49). There are two main proteolytic systems responsible for elaborate implementation of protein degradation: the ubiquitin–proteasome system that is associated with ER quality control of protein folding, and the endosome-lysosomal degradation that safeguards peripheral quality control of membrane-localized proteins (50, 51). Both protein degradation systems are essential for the maintenance of normal cellular functions, and the degradation of a given target protein may involve multiple types of E3 ubiquitin ligases. We have previously demonstrated that the multisubunit E3 ubiquitin ligase CUL7 promotes degradation of both ER-localized immature and plasma-membrane-localized mature Eag1 proteins (17). In light of the current evidence that MKRN1 also mediates ER-associated degradation of the K^+^ channel, herein we suggest a more comprehensive protein homeostasis mechanism for Eag1, as illustrated in the schematic model in Figure 9. We propose that both CUL7 and MKRN1 contribute to ER quality control of immature core-glycosylated Eag1 proteins. In addition, our data are consistent with the idea that MKRN1, but not CUL7, associates with and promotes degradation of nascent, nonglycosylated at the ER. In direct contrast to the role of MKRN1 in exclusively regulating the early stage of Eag1 maturation at the ER, CUL7 is additionally responsible for peripheral quality control by promoting lysosomal degradation of mature, full-glycosylated Eag1 at the cell surface.

The presence of a dual ubiquitination system may ensure efficient removal of misfolded channel proteins, especially for those Eag1 mutants associated with the pathogenesis of the congenital neurodevelopmental anomalies TMBTS and ZLS. Indeed, our biochemical analyses indicate that both CUL7 and MKRN1 contribute to the protein quality control mechanism of ZLS-causing Eag1 mutants (Fig. 8, *B*–*D*) (17). According to previous electrophysiological analyses, the ZLS-causing Eag1 mutant G469R fails to generate detectable K^+^ currents (16). Compared with its WT counterpart, the total protein level of this nonfunctional Eag1 mutant is dramatically decreased (Fig. 8*A*) (17), indicating the presence of impaired protein stability. On the other hand, the ZLS-causing G348R and I467V mutants give rise to functional K^+^ channels with prominent hyperpolarizing shift of the steady-state voltage dependence property (16). Surprisingly, despite the presence of this gain-of-function phenotype on the channel gating property, the K^+^ current amplitude of the G348R and I467V mutants is comparable with that of the WT Eag1 channel (16). This paradoxical observation may be reconciled by our finding that all the aforementioned ZLS-causing Eag1 mutants exhibit substantial reduction in band a/b ratio (Fig. 8*A*), suggesting the presence of sizeable membrane trafficking deficit. Therefore, the current study provides the critical evidence indicating that the pathogenic mechanism of ZLS-causing mutations primarily concerns loss-of-function effect on Eag1 protein homeostasis. Although the mechanistic link between defective Eag1 protein homeostasis and neurodevelopmental anomaly remains unclear, our identification of the dual E3 ubiquitin ligase system may provide future insight to the therapeutic potential of correcting Eag1 protein homeostasis.

## Experimental procedures

### cDNA constructs

cDNAs for rat Eag1 (Dr Olaf Pongs, Saarland University, Germany) and MKRN1 long isoform were subcloned into pcDNA3.1, pcDNA3.1-Myc, or pcDNA3.1-Flag vectors (Invitrogen). Disease-causing (G348R, G469R, and I467V) and glycosylation-deficient Eag1 mutations (N388Q and N406Q; Eag1-QQ), as well as MKRN1 short form and the catalytically inactive E3 ligase mutant MKRN1-H307E, were generated using the QuikChange site-directed mutagenesis kit (Agilent Technologies), followed by verification with DNA sequencing. The other cDNA constructs include pDsRed-Monomer-Membrane (Clontech), pDsRed-ER (Clontech Laboratories), and HA-Ubiquitin (Addgene).

### Cell culture and DNA transfection

Human embryonic kidney 293T (HEK293T) cells were grown in DMEM (Gibco) supplemented with 10% fetal bovine serum (HyClone), 1 mM sodium pyruvate, and penicillin streptomycin at 37 °C incubator with 5% CO_2_. Twenty-four hours before transfection, cells were plated onto 6- or 12-well plates (for biochemical experiments) or poly-D-lysine (Sigma)-coated coverslips in 24-well plates (for immunofluorescence experiments). The amount of Eag1 cDNA used in each well was about 200 (for protein expression) to 1000 (for immunoprecipitation) ng/ml. The molar ratio for cotransfection with MKRN1 was 5:1 (relative to Eag1 cDNA). Transient transfection was performed using the Lipofectamine 2000 reagent (Invitrogen). Various expression constructs were incubated with the transfection reagent for 20 min at room temperature, and DNA-lipofectamine diluted in Opti-MEM (Invitrogen) was added to culture wells. After 6 h of incubation at 37 °C, the medium was changed and transfected cells were maintained in 37 °C incubator for 24 to 48 h. Where indicated, brefeldin A (LC Laboratories; 10 μM), chloroquine (Sigma; 10 μM), cycloheximide (Sigma; 100 μg/ml), or MG132 (Enzo Life Science; 10 μM) was added to the culture medium.

### siRNA and shRNA knockdown

Transfection of small interference RNAs (siRNAs) (Invitrogen), including MKRN1 siRNA (si-MKRN1: 5'-GCATGGAGGTGGTCTATGATT-3’) and negative control (si-Neg: Invitrogen #4390843), was implemented with the Lipofectamine RNAi MAX (Invitrogen). Cells were plated onto 12-well plates 24 h before siRNA transfection. Twenty-four hours after siRNA knockdown, cells were further transfected with Eag1 cDNA. Short hairpin RNAs (shRNAs) in the pLKO.1 puromycin-resistant vector (RNAi Core, Academia Sinica) include MKRN1-shRNAs (sh-MKRN1#1: TRCN0000289977, targeting human and rat MKRN1 sequences; sh-MKRN1#2: TRCN0000289976, targeting human MKRN1 sequence; sh-MKRN1#3: TRCN0000041206, targeting rat MKRN1 sequence) and GFP shRNA (sh-GFP: TRCN0000207020). For lentiviral packaging in HEK293T cells, virus-containing medium was harvested and concentrated using ultracentrifugation (10,000*g* for 4 h at 4 °C) to yield viral stocks. For lentiviral infection in HEK293T cells, viral stocks were supplemented with 8 μg/ml of polybrene (Sigma). Infected HEK293T cells were selected by 5 μg/ml of puromycin (Sigma) and subsequently transfected with Eag1 cDNA. For suppressing endogenous MKRN1 in cultured neurons, 7-day *in vitro* (DIV7) cortical neurons were infected with viral stocks supplemented with 0.4 μg/ml of polybrene. After puromycin selection, infected DIV11 neurons were subject to immunoblotting.

### Immunoprecipitation and immunoblotting

Forty-eight hours after transfection, HEK293T cells were lysed in the ice-cold lysis buffer [(in mM) 100 NaCl, 4 KCl, 2.5 EDTA, 20 NaHCO3, 20 Tris-HCl, pH 7.5, 1 phenylmethylsulfonyl fluoride (PMSF)] with 1% Triton X-100, supplemented with protease inhibitor cocktail (Roche Applied Science). The lysates were cleared by centrifugation at 13,800*g* for 15 min at 4 °C. Protein concentrations were determined by the BCA method (Thermo Scientific). For immunoprecipitation, solubilized lysates were precleared with protein A/G Sepharose beads (GE Healthcare Biosciences) and then incubated for 16 h at 4 °C with protein A/G Sepharose beads precoated with desired antibodies. The beads were washed extensively with the lysis buffer, and the immune complexes were eluted from the beads by boiling for 5 min in Laemmli sample buffer. For glycosidase treatment, solubilized cell lysates were treated with 500 U N-glycosidase F (PNGase F; New England Biolabs) or endoglycosidase H (Endo H; New England Biolabs) for 4 h at 37 °C. Protein samples were separated on SDS-PAGE, transferred onto nitrocellulose membrane (GE Healthcare Biosciences), immunoblotted with appropriate dilution of primary antibodies, and visualized with the ECL detection system (Advansta). Primary antibodies include mouse anti-pan-cadherin (1:2000; Abcam), mouse anti-calnexin (1:2000; Santa Cruz), rabbit anti-Eag1 (1:15,000; Alomone), mouse anti-Flag (1:5000; Sigma), rabbit anti-Flag (1:5000; Sigma), rabbit anti-GAPDH (1:50,000; GeneTex), mouse anti-GFP (1:10,000; Abcam), rat anti-HA (1:5000; Roche), and rabbit anti-MKRN1 (1:5000; Bethyl Laboratories) antibodies. After TBST wash, membranes were incubated with appropriate dilution of horseradish-peroxidase-conjugated goat anti-mouse or goat anti-rabbit secondary antibodies (Invitrogen) for 1 h at RT. Immunoblot signals were quantified by using the ImageQuant software (GE Healthcare Biosciences) and quantified with Image J (National Institute of Health, Bethesda, MD, USA). Results shown are representative of at least three independent experiments.

### Animals and neuronal cultures

All animal procedures were in conformity with the animal protocol approved by the Institutional Animal Care and Use Committee (IACUC) of National Yang-Ming University. Dissociated neuronal cultures were prepared from embryonic embryos (E18 or E19) of Sprague-Dawley rats. Dissociated cortical neurons were plated at 1000 cells/mm^2^. Neurons were maintained in the Neurobasal (Invitrogen) supplemented with 2% B27 (Invitrogen) and 2 mM glutamine in a humidified 5% CO_2_ incubator at 37 °C.

### Immunofluorescence

Cells grown on coverslips were rinsed in ice-cold PBS [(in mM) 136 NaCl, 2.5 KCl, 1.5 KH_2_PO_4_, 6.5 Na_2_HPO_4_, pH 7.4] with 0.9 mM CaCl_2_ and 0.5 mM MgCl_2_ and then fixed for 20 min with 4% paraformaldehyde in PBS at 4 °C. After washing with cold PBS, fixed cells were permeabilized and blocked with a blocking buffer (5% normal goat serum in PBS, 0.05% (v/v) Triton X-100) for 60 min at 4 °C. Cells were then immunolabeled with the following primary antibodies at 4 °C for 16 h: rabbit anti-Eag1 antibody (Alomone; 1:1000). Alexa Fluor 488-conjugated rabbit IgG and Alexa Fluor 568-conjugated mouse IgG (Molecular Probes) were used as secondary antibodies. Cell nuclei were stained with DAPI (1 μg/ml in PBS; Sigma) for 7 min at room temperature. After final wash, coverslips were mounted in a mounting medium (4% n-propyl gallate, 90% glycerol, 0.1 M carbonate buffer, pH 9.2) and observed using a Zeiss LSM880 laser-scanning microscope equipped with 100X oil immersion objective (Zeiss).

To visualize colocalization of Myc-Eag1 and Flag-MKRN1 in HEK293T cells, we employed the Duolink *in situ* proximity ligation assay (Olink Bioscience). After fixation in 4% paraformaldehyde, cells on coverslips were incubated with Duolink blocking buffer for 30 min at 37 °C, followed by immunolabeling with appropriate dilution (in Duolink antibody diluent buffer) of rabbit anti-Flag and mouse anti-Myc antibodies overnight at 4 °C. For negative controls of proximity ligation assay, we applied each primary antibody individually. After being rinsed in Duolink wash buffer A, cells were incubated with the oligonucleotide-labeled Duolink anti-rabbit PLUS and anti-mouse MINUS secondary antibodies (1:5) for 1 h at 37 °C. Samples were then amplified with Duolink detection reagents red for 100 min at 37 °C, followed by rinsing in Duolink wash buffer B, and mounted with Duolink mounting media. Fluorescence images of proximity ligation interaction were acquired using confocal microscopy.

### Preparation of brain homogenates and crude membrane fractions

For crude membrane fraction preparation, rat forebrains were homogenized in buffer H1 [(in mM) 320 sucrose, 1 NaHCO_3_, 0.5 CaCl_2_, 1 PMSF] containing the complete protease inhibitor cocktail (Roche Applied Science). Tissue homogenates were centrifuged at 1400*g* for 10 min to recover the supernatant S1 and the pellet P1. The S1 fraction was subject to centrifugation at 13,800*g* for 10 min to obtain the P2 pellet (crude membrane fractions).

### Glutathione S-transferase (GST) pull-down assay

GST fusion proteins were produced and purified by following the manufacturer’s instruction (Agilent Technologies) as previously reported (17). Briefly, cDNAs encoding the indicated regions of rat Eag1 or MKRN1 were subcloned into the pGEX vector (GE Healthcare, Biosciences) and expressed in the *E. coli* strain BL21. The lysates of IPTG-induced bacteria were incubated with glutathione-agarose beads (Sigma) that bound GST fusion proteins. GST fusion protein-coated beads (4–8 μg) were subsequently incubated overnight with appropriate HEK293T cell lysates or crude membrane fractions from rat cortices at 4 °C and eluted by boiling for 5 min in the Laemmli sample buffer.

### Differential centrifugation and sucrose gradient fractionation

Cells were washed with iced-cold PBS and detached from petri dishes with PBS and 2 mM EDTA. Cells were pelleted for 5 min at 1000*g* and resuspended in hypotonic buffer [(in mM) 20 Tris-HCl, pH 7.9, 2 dithiothreitol, 10 EDTA, and 1 PMSF] supplemented with protease inhibitor cocktail (Roche Applied Science). Cell suspension was homogenized by 20 strokes in a Dounce homogenizer and centrifuged at 1000*g* at 4 °C for 5 min to undisrupted cells. Homogenates were supplemented with 2.0 M sucrose to achieve a 0.2 M final sucrose concentration. After 90 min centrifugation at 100,000*g*, supernatant and pellet fractions contained cytosolic and membrane proteins, respectively. Pellets were rinsed with hypotonic buffer and resuspended to the same volume as supernatants in iced-cold lysis buffer [(in mM) 100 NaCl, 4 KCl, 2.5 EDTA, 20 NaHCO_3_, 20 Tris-HCl, pH 7.5, 1 PMSF] with 1% Triton X-100. For sucrose gradient fractionation, we employed a previously published protocol with some modifications (17). Cells were lysed and ultracentrifuged to separate the cytosol and the membrane fractions as describe above. Membrane pellet fractions were suspended in hypotonic buffer supplemented with 2.0 M sucrose to achieve a 0.2 M final sucrose concentration. Membrane lysates were loaded on the top of a discontinuous sucrose gradient [2.0 M (0.4 ml), 1.5 M (0.75 ml), 1.35 M (0.75 ml), 1.2 M (0.75 ml), 0.9 M (0.5 ml), and 0.5 M (0.5 ml) in hypotonic buffer] and subject to a 32,000*g* centrifugation for 16 h in a Beckman SW55 rotor. Fractions were collected from the top of the gradient, including one 1.0-ml and seven 0.5-ml fractions.

### Mass spectrometry analysis and protein identification

Three 10-cm petri dishes of HEK293T cells (∼1.5 × 10^7^ cells per dish) were transfected with the Eag1 plasmid. Forty-two hours after transfection, cells were treated with 10 μM MG132 for 6 h to induce the accumulation of Eag1 band c, followed by immunoprecipitation with the anti-Eag1 antibody. The immunoprecipitates were eluted with 150 μl 0.5% SDS, 40 mM DTT at 95 °C for 15 min. One-third of the eluent (50 μl) was digested with PNGase F at 37 °C for 4 h. The eluents with or without PNGase F digestion were concentrated with a centrifugal filter at 10,000*g* for 20 to 40 min. Eluted complexes were resuspended in 2X Laemmli buffer and subject to SDS-PAGE separation for immunoblotting or silver staining. Gel sections of Eag1 bands a, b, c, and PNGase F-treated Eag1 (band d) were diced into small pieces (1 × 1 mm). Each gel piece was placed into a 2-ml tube and dehydrated with 25 mM NH_4_HCO_3_ and 50% (v/v) acetonitrile (1:1). After drying in a Speed-Vac (Thermo Electron), gel pieces were rehydrated with 25 mM NH_4_HCO_3_ and 1% β-mercaptoethanol in dark for 20 min at 56 °C, followed by incubation in a solution containing 5% 4-vinylpyridine in 25 mM NH_4_HCO_3_ and 50% acetonitrile (1:1) for 20 min at room temperature. Protein digestions were performed with trypsin in 10 μl formic acid (0.1%, v/v) at 37 °C overnight. Digested proteins were then desalted for nanoscale liquid chromatography coupled to tandem mass spectrometry (nano-LC-MS/MS) analysis, using a nanoAcquity system (Waters) coupled to an Orbitrap Velos hybrid mass spectrometer with a nanoelectrospray ionization source (ThermoFisher Scientific), at the Metabolomics-Proteomics Research Center, National Yang-Ming University, Taiwan. Peptides for analysis were loaded *via* a BEH C_18_ column (length: 25 cm; inner diameter: 75 μm; Waters) and separated at a flow rate of 300 nl/min using mobile phase acetonitrile with the addition of 0.1% formic acid in a gradient of 5 to 35% over 120 min. Eluted peptides were examined with the LTQ-Orbitrap mass spectrometer using a spray voltage of +1.7 kV. The mass spectrometer was conducted in the positive ion mode and on the basis of a data-dependent acquisition method (isolation width: 2.0 Da). The resolution of full MS was set to 30,000 with the m/z range of 400. Mass spectrum data of the peptides were obtained using a full spectrometer survey scan (m/z range of 350–1600). According to the data-dependent acquisition method, the ten most intense multiply charged ions (2^+^ and 3^+^) were selected for MS/MS scan. Collision-induced dissociation was confirmed for MS/MS. The acquired MS/MS data were analyzed by Peaks7.5 Studio software for Proteomics (Bioinformatics Solutions) *via* searching the UniProt rat protein database (UniProt database: Rat-UP-(Unreviewed)-2021-01-06-and-Eag1.fasta; number of entries searched in the database: 28,068). Search parameters were set as follows: enzyme, trypsin; precursor ion mass tolerance, 50 ppm; fragment ion mass error tolerance, 0.8 Da; precursor mass search type, monoisotopic; maximum missed cleavages allowed 2; and oxidation on methionine (+15.99 Da) and S-pyridylethylation on cysteine (+105.06 Da) for variable modifications. The average local confidence was established as over 80%. A decoy database was used to calculate the false discovery rate, which was set as below 0.1%. A protein was identified when at least one unique peptide was matched.

### Electrophysiology

Whole-cell voltage clamp recordings of Eag1 K^+^ currents in HEK293T cells were performed 24 h posttransfection. Electrode solution contains (in mM) 140 KCl, 1 MgCl_2_, 10 EGTA, 10 HEPES, pH 7.2. Bath solution comprises (in mM) 140 NaCl, 5 KCl, 1 CaCl_2_, and 10 HEPES, pH 7.2. Data were acquired with the Axopatch 200B amplifier (Molecular Devices) and digitized with the Digidata 1440A system and the pCLAMP 10.2 software (Molecular Devices). Cells with large currents in which voltage clamp errors might appear were excluded from data analyses. Passive membrane properties were compensated by using the -P/4 leak subtraction method. Data were sampled at 10 kHz and filtered at 1 kHz. All recordings were performed at room temperature (20–22 °C).

### Statistical analysis

Data are presented as mean ± SD. The significance of the difference between two means was tested by Student’s *t*-test, whereas means from multiple groups were compared by one-way ANOVA. All statistical analyses and curve fitting were performed with the Origin 7.0 (Microcal Software) or Prism 6 (GraphPad Software).

## Data availability

Information on identified peptides and their charge states has been submitted to ProteomeXchange. Data are available *via* ProteomeXchange with identifier PXD023984 (https://doi.org/10.6019/PXD023984).

## Supporting information

This article contains supporting information.

## Conflict of interests

The authors declare that they have no conflicts of interest with the contents of this article.
