# Supplementary material for: Identification of MKRN1 as a second E3 ligase for Eag1 potassium channels reveals regulation via differential degradation
Source: J Biol Chem. 2021 Feb 27;296:100484. doi: 10.1016/j.jbc.2021.100484 (PMC8039722; doi:10.1016/j.jbc.2021.100484)
Supplement: Supplemental Figures S1–S6 [file mmc1.pdf]

Identification of MKRN1 as a second E3 ligase for Eag1 potassium channels reveals regulation via differential degradation

Ya-Ching Fang, Ssu-Ju Fu, Po-Hao Hsu, Pei-Tzu Chang, Jing-Jia Huang, Yi-Chih Chiu, Yi-Fan Liao, Guey-Mei Jow, Chih-Yung Tang, and Chung-Jiuan Jeng

Supporting Table and Figures

Supporting Table S1.

An Excel file that reports the detailed mass spectrometry information for all peptide sequences assigned for each Eag1 protein bands in Supporting Figure S2.

Supporting Figure S1.

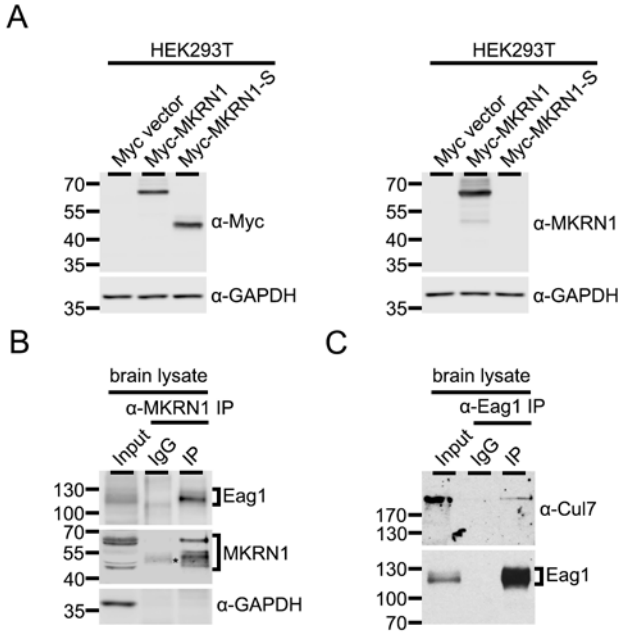

Supporting Figure S1. Association of endogenous MKRN1 / CUL7 with Eag1 in neurons. (A) Verification of the specificity of the anti-MKRN1 antibody. Lysates from HEK293T cells over-expressing the Myc vector, Myc-MKRN1, or Myc-MKRN1-S were immunoblotted with anti-Myc (*left*) or anti-MKRN1 (*right*) bodies. The commercially available anti-MKRN1 antibody (Bethyl Laboratories A300-990A) only detects the long, but not the short, isoform of MKRN1. According to the manufacturer's data sheet, the immunogen of the anti-MKRN1 antibody is located between amino acid 432 and the C-terminus of MKRN1, which is not found in the short isoform. (B-C) Immunoprecipitation experiments with rat brain lysates were performed using anti-MKRN1 (B) or anti-Eag1 (C) antibodies, followed by immunoblotting with the indicated antibodies. Rabbit IgG was also employed for immunoprecipitation as a control. In both HEK293T cells and neurons, the anti-MKRN1 antibody detects multiple MKRN1-specific bands (putative endogenous splice variants) at 45~65 kDa.

Supporting Figure S2.

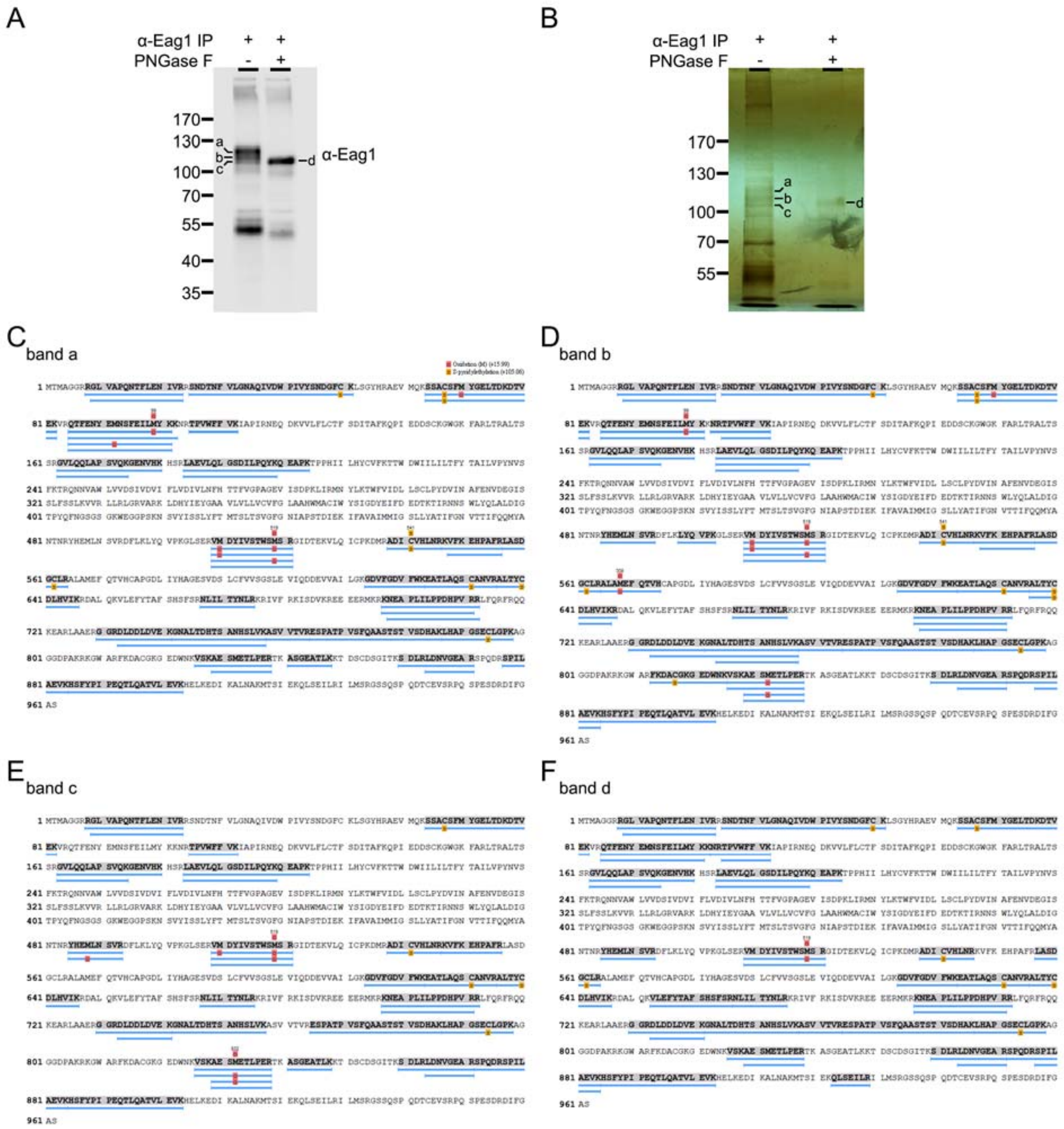

Supporting Figure S2. Mass spectrometry analyses of protein bands. (A-B) Representative Eag1 protein bands. Lysates from MG132-treated HEK293T cells over-expressing rat Eag1 were subject to immunoprecipitation with the anti-Eag1 antibody. Eluted immunoprecipitates were divided into two fractions, one with PNGase F treatment and the other as the no-treatment control. Both fractions were then separated by SDS-PAGE and detected with immunoblotting (A) or silver staining (B). As exemplified in Figures 2 and 5, three distinct protein bands with Eag1-comparable molecular weights are noted in the no-treatment control samples (bands a, b, and c), whereas the PNGase F-treated samples yield a single band labeled here as band d. (C-F) Representative peptide coverage maps for Eag1 bands. Gel sections harboring bands a, b, c, and d were diced into small pieces, followed by in-gel digestion with trypsin. The resulting peptides were then desalted for analysis with nano-LC-MS/MS to verify protein identities. Among the top hits with a false

discovery rate less than 0.1%, Eag1 is predicted to be the best-matched unique peptide. Blue lines: peptides generated by trypsin digestion. Grey highlights: Eag1 amino acid sequences matched by trypsin-digested peptides. Oxidation: oxidation on methionine. S-pyridylethylation: S-pyridylethylation on cysteine. Eag1 sequence coverage rate: band a, 37% (a total of 36 peptides); band b, 41% (43 peptides); band c, 32% (30 peptides); band d, 39% (37 peptides). These data are representative of two independent experiments. For all peptide sequences assigned for each Eag1 protein bands here, we have provided an Excel file (Supporting Table S1) that reports the following information: list of all peptide sequences identified, precursor charge and m/z for each assignment, modifications observed, sites of modification within each peptide clearly located, and peptide identification scores.

Supporting Figure S3.

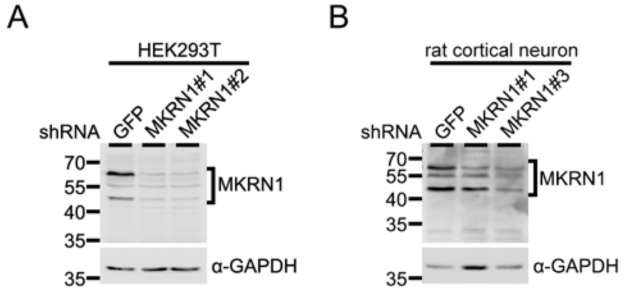

Supporting Figure S3. Verification of the effect of shRNA knockdown of endogenous MKRN1 expression. HEK293T cells (A) or rat cortical neurons (11 days *in vitro*) (B) were subject to viral infection with the indicated shRNA constructs, followed by immunoblotting with ant-MKRN1 and anti-GAPDH antibodies.

Supporting Figure S4.

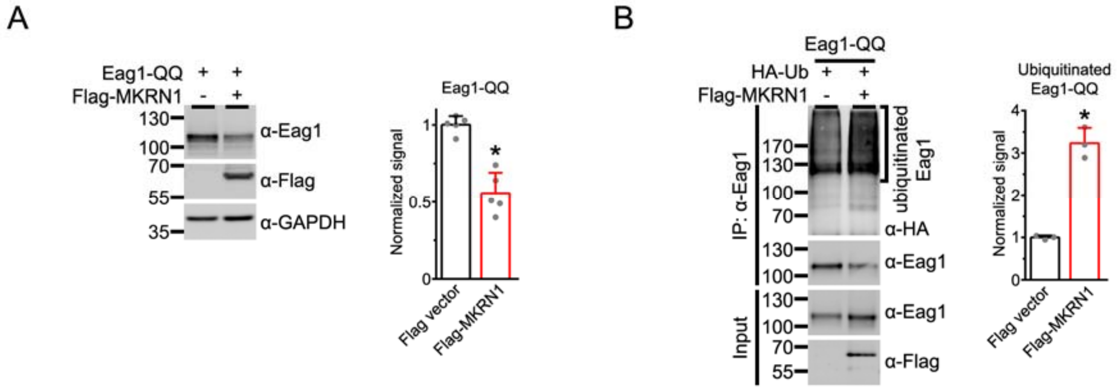

Supporting Figure S4. MKRN1 regulates protein homeostasis of the Eag1-QQ mutant. Effect of MKRN1 co-expression on protein level (A) and HA-Ub-mediated ubiquitination (B) of Eag1-QQ in HEK293T cells. Eag1-QQ was co-transfected with Flag-MKRN1 in the molar ratio 1:5. Quantitative analyses are based on normalization with respect to the Flag vector control (*, $P<0.05$; n=3-5).

Supporting Figure S5.

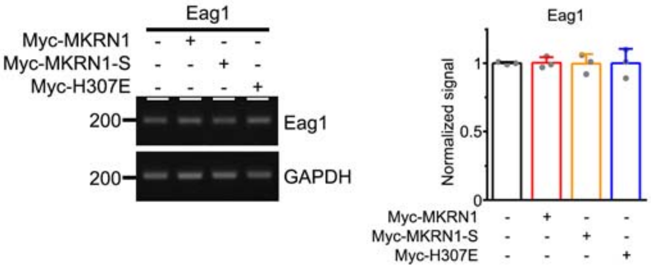

Supporting Figure S5. Lack of effect of MKRN1 on Eag1 mRNA levels. Semi-quantitative reverse transcription-PCR (RT-PCR) analyses of Eag1 mRNA expressed in HEK293T cells. Eag1 was co-expressed with Myc vector (-), Myc-MKRN1 long-form (Myc-MKRN1), Myc-MKRN1 short-form (Myc-MKRN1-S), or Myc-MKRN1-H307E (Myc-H307E) in HEK293T cells. mRNA levels of Eag1 were standardized as the ratio of Eag1 signals to the GAPDH mRNA levels, followed by normalization to those of the Myc vector control (n=3).

Supporting Figure S6.

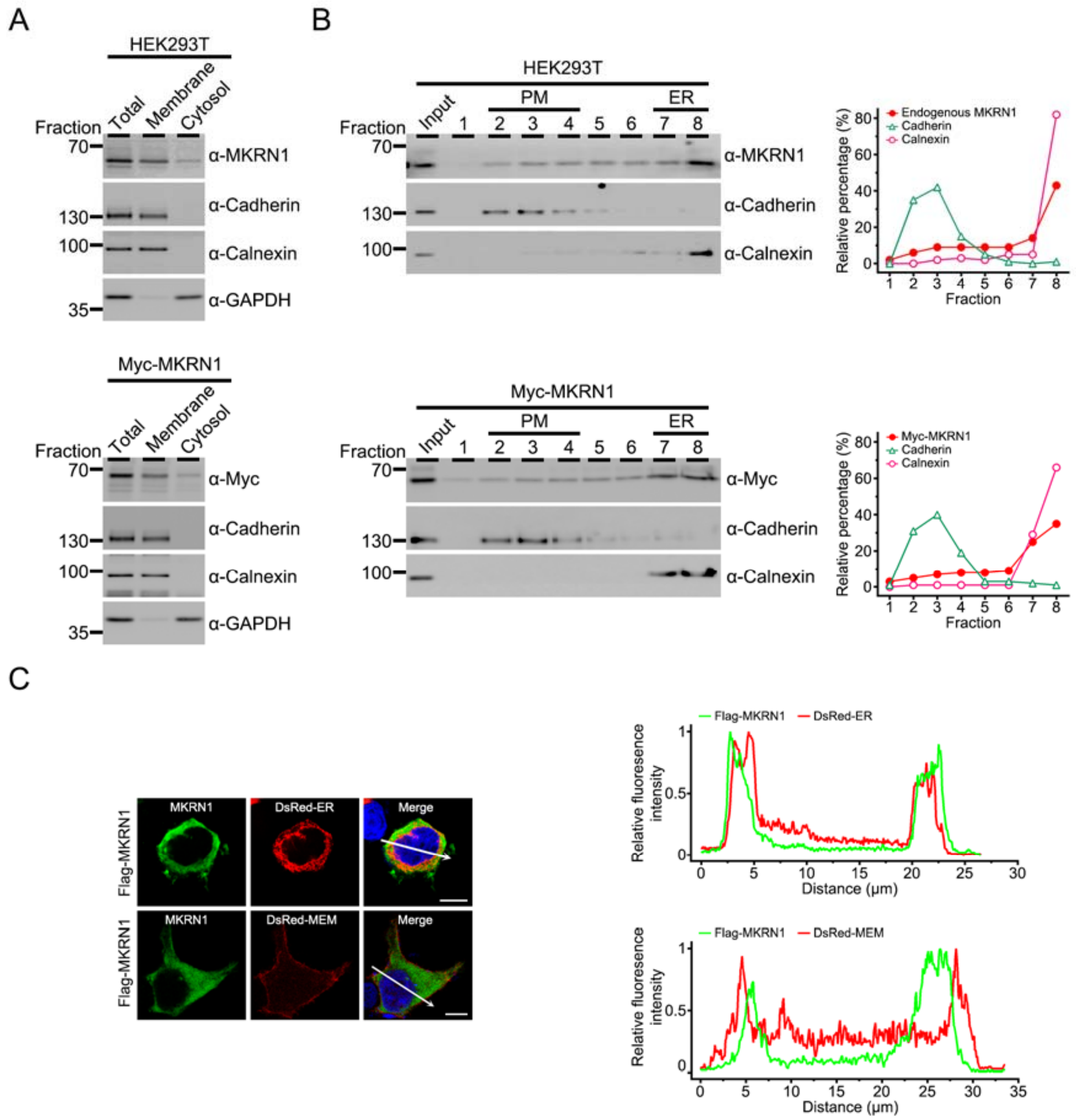

Supporting Figure S6. Localization of MKRN1 at the ER membrane. (A) Differential centrifugation analyses of endogenous (*top*) and over-expressed (*bottom*) MKRN1 in HEK293T cells. Total cell homogenates were ultra-centrifuged and thereafter separated into the membrane and the cytosol fractions. Also shown are the centrifugation pattern of the plasma membrane-localized cadherin and the ER membrane-resident calnexin. (B) Sucrose gradient fractionation analyses of endogenous (*top*) and over-expressed (*bottom*) MKRN1 in HEK293T cells. (*Left panels*) Representative immunoblots. The membrane pellet fraction from differential centrifugation analyses was further sedimented through a discontinuous sucrose gradient and subsequently divided into 8 fractions, with the density gradient increasing from fraction 1 toward fraction 8. (*Right panels*) Densitometric quantification of the relative distribution (with respect to the total signal) of the indicated proteins in each membrane-associated fraction. (C) (*Left panels*) Representative immunofluorescence images showing the subcellular localization of Flag-MKRN1 (*green*) over-expressed in HEK293T cells co-transfected with the ER marker DsRed-ER (*red*; *top*), or the

plasma membrane marker DsRed-Membrane (*DsRed-MEM; red; bottom*). Nuclei were counterstained with DAPI (*blue*). Scale bar, 10 µm. Data are representative of at least 3 independent experiments. (*Right panels*) Quantitative analyses of the co-localization of Flag-MKRN1 (*green*) and DsRed-ER (*red*; *top*) or DsRed-MEM (*red; bottom*) fluorescence signals. Relative fluorescence intensities along the white arrows traversing the indicated cells (*left panels*) were compared to highlight the notion that the majority of the MKRN1 fluorescence signals co-localize with those of DsRed-ER, but not DsRed-MEM.
